# Anion Recognition by a Bioactive Diureidodecalin Anionophore: Solid‐State, Solution, and Computational Studies

**DOI:** 10.1002/chem.201800537

**Published:** 2018-05-14

**Authors:** Ondřej Jurček, Hennie Valkenier, Rakesh Puttreddy, Martin Novák, Hazel A. Sparkes, Radek Marek, Kari Rissanen, Anthony P. Davis

**Affiliations:** ^1^ Department of Chemistry University of Jyvaskyla P.O. Box 35 40014 Jyväskylä Finland; ^2^ School of Chemistry University of Bristol Cantock's Close Bristol BS8 1TS UK; ^3^ CEITEC—Central European Institute of Technology Masaryk University Kamenice 5/A4 Brno Czech Republic; ^4^ Engineering of Molecular NanoSystems, Ecole Polytechnique de Bruxelles Université Libre de Bruxelles Avenue F.D. Roosevelt 50, CP165/64 1050 Brussels Belgium; ^5^ Department of Chemistry, Faculty of Science Masaryk University Kamenice 5/A4 625 00 Brno Czech Republic

**Keywords:** anions, host–guest interactions, hydrogen bonds, receptors, solid-state structures

## Abstract

Recent work has identified a bis‐(*p*‐nitrophenyl)ureidodecalin anion carrier as a promising candidate for biomedical applications, showing good activity for chloride transport in cells yet almost no cytotoxicity. To underpin further development of this and related compounds, a detailed structural and binding investigation is reported. Crystal structures of the transporter as five solvates confirm the diaxial positioning of urea groups while revealing a degree of conformational flexibility. Structures of complexes with Cl^−^, Br^−^, NO_3_^−^, SO_4_^2−^ and AcO^−^, supported by computational studies, show how the binding site can adapt to accommodate these anions. ^1^H NMR binding studies revealed exceptionally high affinities for anions in DMSO, decreasing in the order SO_4_^2−^>H_2_PO_4_^−^≈HCO_3_^−^≈AcO^−^≫HSO_4_^−^>Cl^−^>Br^−^>NO_3_^−^>I^−^. Analysis of the binding results suggests that selectivity is determined mainly by the H‐bond acceptor strength of different anions, but is also modulated by receptor geometry.

## Introduction

Synthetic anion receptors and carriers (anionophores) have attracted great attention from supramolecular chemists over the last few decades.^1^ An important motivation is the potential for applications in biology and medicine, resulting from the promotion of anion transport across cell membranes. There is particular interest in developing treatments for diseases resulting from dysfunctional anion channels, which could be bypassed by using synthetic transporters. Such “channelopathies” include Best disease, Startle disease, Bartter's syndrome and, most notably, the widespread genetic disease cystic fibrosis (CF).^2^

**Figure 1:**
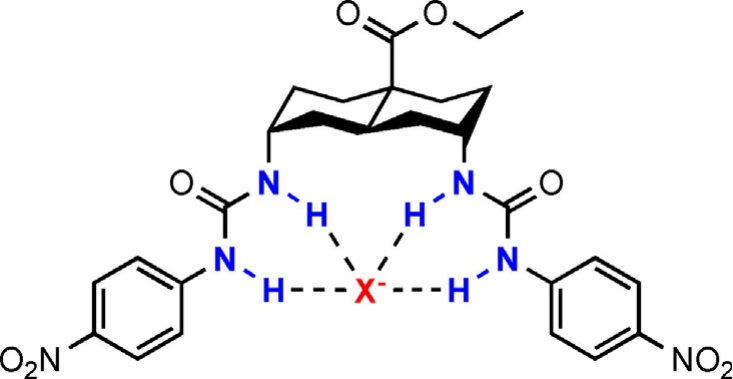
Transporter **1** binding an anion X^−^ (red), where X^−^=SO_4_^2−^, HSO_4_^−^, H_2_PO_4_^−^, HCO_3_^−^, NO_3_^−^, AcO^−^, Cl^−^, Br^−^ or I^−^. H‐bond donor NH groups are highlighted in blue.

While research in this area is largely driven by biology, the majority of studies have involved synthetic membrane systems (typically large unilamellar vesicles). There are still few examples of anionophores with proven effectiveness in cells.^3^ In a recent report, Davis et al. described studies employing epithelial cells engineered to express yellow fluorescent protein (YFP).^4^ The fluorescence of YFP is halide‐sensitive, so can be used to monitor the transport of halide anions across cell membranes. The anionophores used in this work belonged to the “1,5‐diaxial” family.^5^ These carriers employ binding sites created by H‐bond donor groups, especially (thio)ureas,^6^ positioned in *cis*‐1,5 relationships on alicyclic scaffolds (steroids,^7^ *trans*‐decalins^8^, ^9^ and cyclohexanes).^10^ Among the compounds tested, diureidodecalin **1** (Figure 1) was especially active. It induced rapid chloride transport, on a timescale of a few minutes, and appeared to persist in the cell membrane for a period of hours. When further tested in an epithelium mounted in an Ussing chamber, low levels were found to support significant currents. The implied rates of chloride transport came quite close to matching those due to CFTR, the anion channel associated with CF. Transporter **1** showed almost no cytotoxicity in various cell lines, and therefore seems a promising candidate for treatment of channelopathies.

The development of **1** for biomedical applications will require a full understanding of its behaviour. While anion transport by diureidodecalins has been studied quite intensively, less is known about their structural and binding properties. In particular, whereas binding to chloride is well‐characterised, including a crystal structure of the **1⋅**NMe_4_^+^Cl^−^ complex,^4^ the complexation of other anions has received less attention. Here we report an investigation of **1** as a receptor for various biogenic anions, considering both structures and affinities. We describe crystal structures of **1** itself as well as four new complexes, supplemented by computational studies. We also report binding experiments to nine anions, and apply a simple computational aid to gain insight into selectivity.

## Results and Discussion

### Single‐crystal X‐ray crystallographic studies on 1

**Figure 2:**
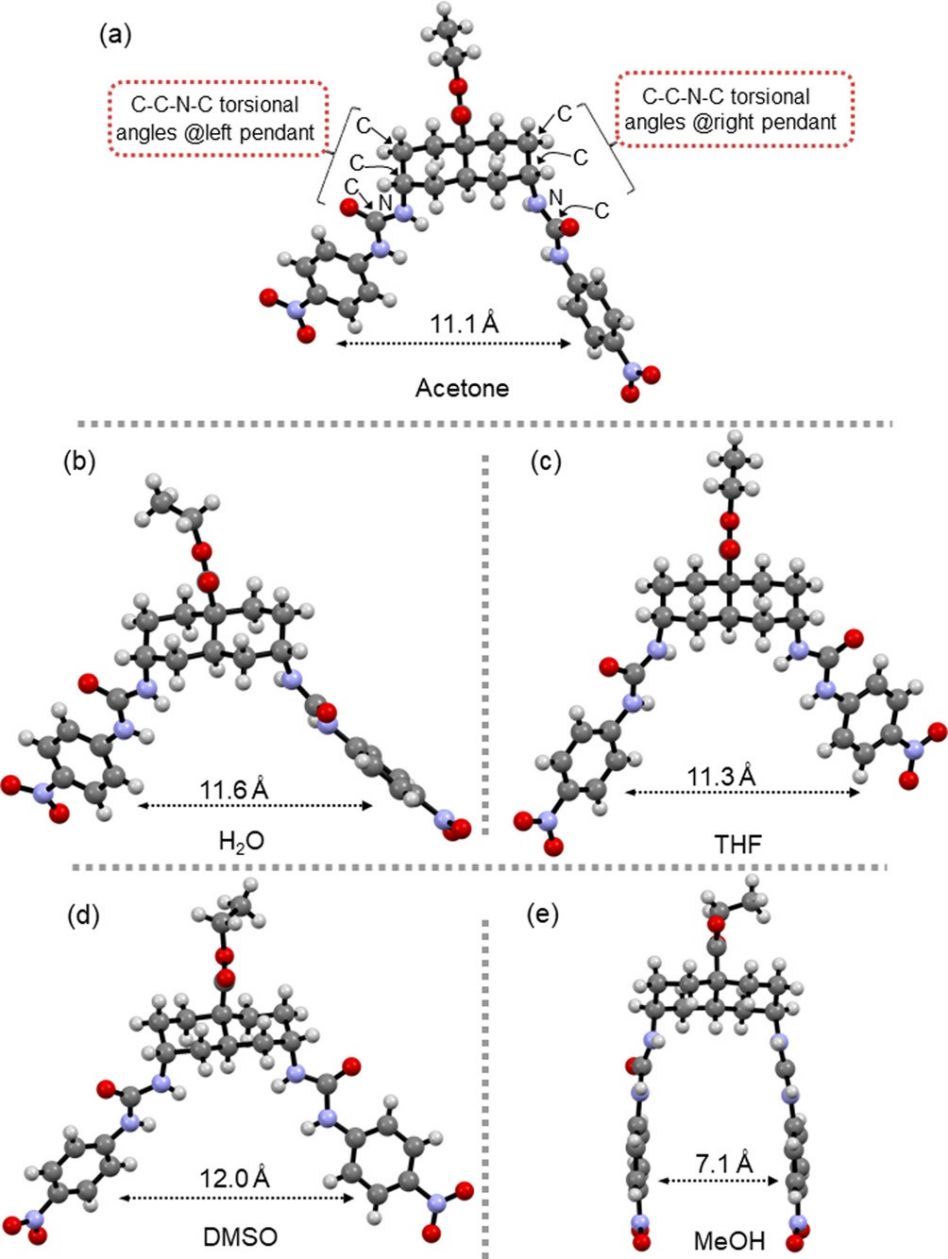
X‐ray structures of transporter **1** solvates. a) Acetone, b) H_2_O, c) THF, d) DMSO and e) MeOH. The aromatic centroid‐to‐centroid distance is shown for each structure.

Urea and thiourea derivatives are well known for their ability to form H‐bonds with molecules containing H‐bond acceptors, such as solvents and anions. Transporter **1** was synthesised as reported previously^4^ and single crystals suitable for X‐ray crystallography were obtained from various solvents or solvent mixtures by slow evaporation. Transporter **1** adopts two distinct conformations in the solid‐state, differentiated by the torsion angles of the planar ureido groups towards the decalin plane (Table 1). When crystallised from acetone, H_2_O,^11^ THF and DMSO, **1** adopts an “open” conformation (Figure 2 a–d). For the acetone, H_2_O and THF solvates the torsion angles of the left and right ureido arms (as viewed in Figure 2) are in the ranges 69–78° and 132–145° respectively. This conformation results from the interaction of the nitro group of the adjacent transporter molecule with one or both of the urea moieties of the transporter **1** (see Supporting Information, Figures S1–S3). In the DMSO solvate the left dihedral angle is of similar value, 81°, whereas the right dihedral changes to 93°. The DMSO molecules are H‐bonded to the urea moieties of transporter **1** (see Supporting Information, Figure S4) in a similar fashion to the anions studied (see below). Aromatic centroid‐to‐centroid distances between the two ureido arms range from about 11 to 12 Å (see Supporting Information, Figure S5). The MeOH solvate (Figure 2 e) is completely different, with respective torsion angles of 165 and 161°. This leads to a nearly parallel orientation of the ureido arms and a “closed” clip‐like molecular conformation with much shorter centroid‐to‐centroid distance of 6.7 Å. The exceptional closed conformation of the MeOH solvate results from strong MeOH‐mediated H‐bonding between the carbonyl oxygen and the urea NH atoms, twisting the ureido groups to nearly parallel orientation (see Supporting Information, Figure S6). These crystal structures show a large degree of freedom of rotation of the urea groups around the C−N bonds which connect them to the decalin scaffold, giving **1** the potential to act as host for a range of different anions.

**Table 1:** C‐C‐N‐C torsion angles between the decalin and ureido groups in the solvates of transporter **1**, giving **1** the potential to act as host for a range of different anions, as also indicated here and discussed below.^[a]^

Solvate or complex of **1**	C‐C‐N‐C torsion angles in the left ureido group	C‐C‐N‐C torsion angles in the right ureido group
acetone	−72/+72	+145/−145
H_2_O	−78/+78	+132/−132
THF	−69/+69	+134/−134
DMSO	−81/+81	+93/‐93
MeOH	−165/+165	+161/−161
**1⋅Cl^−^**	−112/+112	+148/−148
**1⋅Br^−^**	−69/+69	+89/−89
**1⋅AcO^−^**	−91/+91	+77/−77
**1⋅NO_3_** ^**−**^	−68/+68	+83/−83
**1⋅SO_4_** ^**2−**^	−75/+75	+100/−100

[a] Due to the centrosymmetric space group two inverted sets of torsion angles apply: −72, +145 and +72, −145.

### Solid‐state and computational studies on 1⋅X^−^ complexes

Single crystals suitable for X‐ray analysis of complexes **1⋅X^−^** (**X^−^**=Cl^−^, Br^−^, AcO^−^, NO_3_^−^ and SO_4_^2−^) were obtained by slow evaporation of the solvents, with Me_4_N^+^, Et_4_N^+^, or Bu_4_N^+^ as counter cations (for details, see Supporting Information). The distances between urea nitrogen atoms and anions are listed in Table 2. Crystal structures were not obtained for all the anions of interest, so computational studies were performed to analyse the remaining systems. Models of **1** and its complexes with anions were optimized by DFT in Turbomole, initially in vacuum and then in DMSO solvent by using an implicit COSMO model (for computational details, see Supporting Information and Experimental Section). For modelling we used the X‐ray crystal structure of **1⋅Br^−^** for spherical anions, **1⋅NO_3_** ^**−**^ for trigonal complexes and **1** from **(1)_2_⋅SO_4_** ^**2−**^ for the tetrahedral complexes.

**Table 2:** Binding and structural data for complexes of **1** with anions.

Complex	Binding constants from NMR titrations *K*_a_ [m ^−1^]^[a]^	DFT binding energy [kcal mol^−1^]		Literature Δ*G*, anion hydration [kcal mol^−1^]^12^	Urea nitrogen–anion distances [Å]^[e]^	
		Receptor^[c]^	Single arm^[d]^		Crystal structure	Molecular model
**1⋅I^−^**	<5	−16.1	−6.3 (−12.6)	−65.7	–	3.68 (2), 3.63 (2)
**1⋅NO_3_** ^**−**^	10	−17.7	−8.5 (−17.0)	−71.7	3.00, 3.11, 2.91, 2.98	3.07 (2), 2.89 (2)
**1⋅Br^−^**	70	−16.6	−7.0 (−14.0)	−75.3	3.52, 3.48, 3.54, 3.44	3.48 (2), 3.42 (2)
**1⋅HSO_4_** ^**−**^	180	−17.7	−8.2 (−16.4)	–	–	2.96 (2), 2.90, 2.91
**1⋅Cl^−^**	670	−16.9	−7.7 (−15.4)	−81.3	3.30, 3.43, 3.27, 3.20	3.32 (2), 3.25, 3.22
**1⋅AcO^−^**	40 000	−22.8	−13.1 (−26.2)	−87.2	2.86 (2), 2.80, 2.74	2.81 (2), 2.76, 2.78
**1⋅HCO_3_** ^**−**^	42 000	−20.2	−11.7 (−23.4)	−80.1	–	2.98, 3.01, 2.93, 2.79
**1⋅H_2_PO_4_** ^**−**^	46 000	−22.4	−9.2 (−18.4)	−111.1	–	2.88, 2.86, 2.81, 2.80
**1⋅SO_4_** ^**2−**^	>10^5 [b]^	−27.4	−12.9 (−25.8)	−258.1	2.92, 2.93 (2), 2.87 (2), 2.94, 3.01, 3.00	2.90 (2), 3.01 (2), 2.98 (2), 3.30, 3.54
**1⋅HPO_4_** ^**2−**^	–	−30.6	−15.2 (−30.4)	–	–	2.80, 2.75, 2.72, 2.69

[a] [D_6_]DMSO/H_2_O (200:1). [b] Weaker binding of a second sulfate anion forming **1⋅(SO_4_** ^**2−**^ **)_2_** was observed. [c] The binding energy was obtained by subtracting the total energies of the optimised anion and transporter from that of the anion–transporter complex in DMSO. [d] The binding energy was obtained by subtracting the total energy of the optimised anion and single arm [methyl(*p*‐nitrophenyl)urea] from that of the anion–arm complex in DMSO. Doubling these energies gives the values in parentheses, for comparison to the values for the preorganised receptor. [e] Distances from nitrogen atoms to halide anions, or to the nearest oxygen atom of oxoanions. Numbers in brackets refer to the multiplicity of identical distances to different nitrogen atoms.

### Conformations of transporter 1 with halides

**Figure 3:**
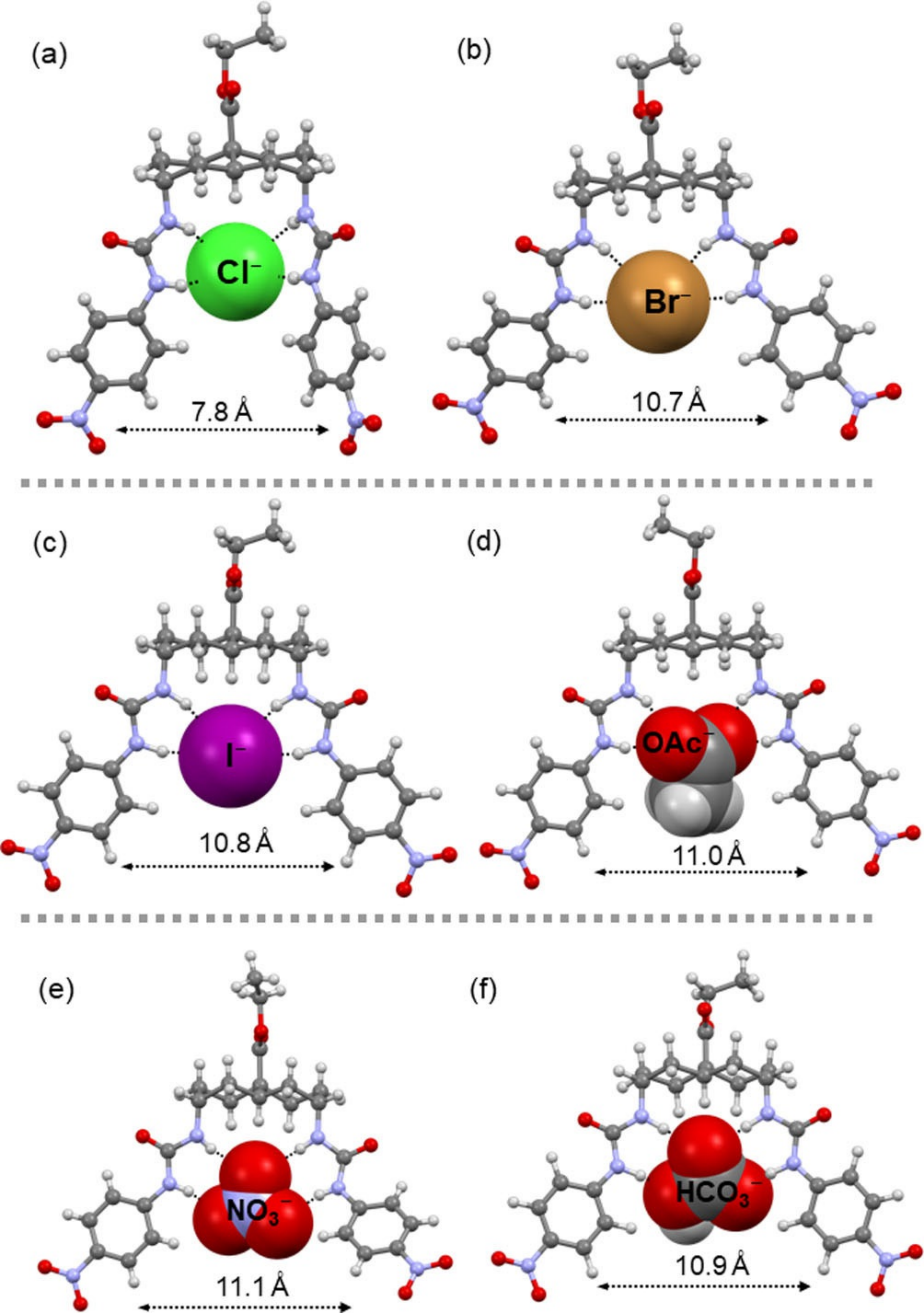
X‐ray crystal structures of a) **1⋅Cl^−^**,^4^ b) **1⋅Br^−^**, d) **1⋅AcO^−^** and e) **1⋅NO_3_** ^**−**^. Energy‐minimised models of c) **1⋅I^−^** and f) **1⋅HCO_3_** ^**−**^. The transporter is shown in ball‐and‐stick mode, and the anions in CPK mode. The countercations are omitted for clarity. The aromatic centroid‐to‐centroid distance is shown for each structure.

Crystal structures of **1⋅Cl^−^** and **1⋅Br^−^**, as well as the DFT optimized structure of **1⋅I^−^**, are shown in Figures 3 a–c. In **1⋅Cl^−^**, three urea NH groups show H‐bonds to the chloride anion at N**⋅⋅⋅**Cl distances of 3.20, 3.27 and 3.30 Å, while the fourth H‐bond is longer than the sum of van der Waals distances (3.43 Å). The framework of transporter **1** in **1⋅Cl^−^** is intermediate between open and closed conformations with an aromatic centroid‐to‐centroid distance of 7.78 Å. In **1⋅Br^−^**, the anion displays one short contact with N**⋅⋅⋅**Br distance of 3.44 Å, while the three other distances are ≥3.50 Å. In the calculated structure of **1⋅I^−^** the N**⋅⋅⋅**I distances are >3.63 Å. In **1⋅Br^−^** and **1⋅I^−^**, to accommodate larger anions, the transporter framework adopts the open conformation with aromatic centroid‐to‐centroid distances of 10.67 and 10.77 Å, respectively.

### Conformations of transporter 1 with planar oxoanions

The delocalisation of negative charge in acetate oxygen atoms induces partial single‐bond character over two C−O bonds [1.246(5) and 1.252(6) Å]. Therefore, in **1⋅AcO^−^**, each oxygen atom acts as a bidentate acceptor for NH groups forming two bifurcated H‐bonds with N**⋅⋅⋅**O distances ranging between about 2.74 and 2.86 Å, as shown in Figure 3 d. The aromatic centroid‐to‐centroid distance is 10.96 Å. The acetate anion is twisted away from the plane of the urea groups, and this possibly implies that the anion is slightly too large for the binding site (see discussion below). The situation differs significantly upon exchange of the acetate methyl group for a third H‐bond acceptor, as in the case of NO_3_^−^ or HCO_3_^−^. The nitrate anion in **1⋅NO_3_** ^**−**^ is positioned such that all the O atoms are involved in H‐bonding, as shown in Figure 3 e. Its triangular shape fits precisely into the binding site of the receptor. A similar conformation was observed in the DFT optimized structure of **1⋅HCO_3_** ^**−**^ (Figure 3 f). The transporter in **1⋅NO_3_** ^**−**^ and **1⋅HCO_3_** ^**−**^ has aromatic centroid‐to‐centroid distances of 11.25 and 10.90 Å, respectively. The N**⋅⋅⋅**O distances in **1⋅NO_3_** ^**−**^ lie between 2.91 and 3.11 Å, while for **1⋅HCO_3_** ^**−**^ they are within 2.89–3.07 Å, that is, nearly in the same range.

### Geometries of complexes with tetrahedral oxoanions

**Figure 4:**
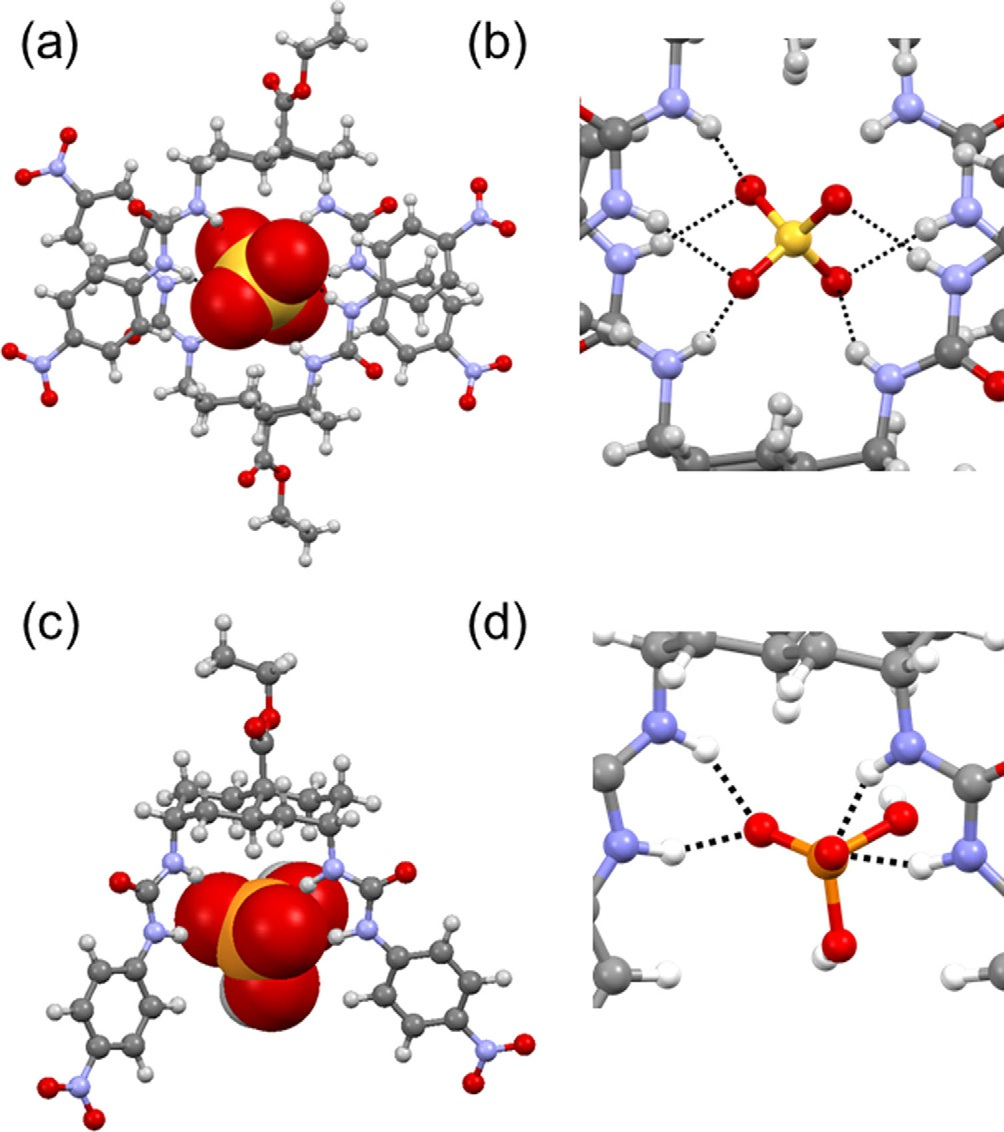
a) X‐ray crystal structure of **(1)_2_⋅SO_4_** ^**2−**^. The transporter is shown in ball‐and‐stick mode, and the anion in CPK mode. b) A closer view of the H‐bonds around the sulfate anion in the dimer. The Me_4_N^+^ cation and solvent molecule are omitted for clarity. c) DFT model of **1⋅H_2_PO_4_** ^**−**^. d) A closer view of the H‐bonds around the dihydrogenphosphate anion. H‐bonds are shown as black dashed lines.

Interestingly, the crystallisation of **1⋅SO_4_** ^**2−**^ provided us with a structure in which two molecules of **1** share one molecule of SO_4_^2−^ [**(1)_2_⋅SO_4_** ^**2−**^, Figure 4 a]. This may reflect the strong affinity of **1** towards doubly charged SO_4_^2−^, as well as the ability of this large anion to accommodate two bis‐urea receptors in its coordination sphere. Similar (receptor)_2_ **⋅**Cl^−^ structures have previously been suggested for a bis(phenylureido)decalin transporter in chloroform.^8^ In the crystal structure of **(1)_2_⋅SO_4_** ^**2−**^, all four oxygen atoms of the sulfate anion are involved in H‐bonding to seven urea NH groups (Figure 4 b) with N**⋅⋅⋅**O distances ranging between about 2.87 and 3.00 Å. Within the receptor framework, the aromatic rings are separated at centroid‐to‐centroid distances of about 11.4 Å and show weak π–π contacts with shortest C**⋅⋅⋅**C distances of about 3.30 Å (see Supporting Information, Figure S11).

Attempts to crystallise **1⋅H_2_PO_4_** ^**−**^ were unsuccessful, so insight into the binding was obtained by DFT calculations (Figure 4 c, d). In the model, each urea unit binds to one oxygen atom through H‐bonds with N**⋅⋅⋅**O distances ranging between 2.80 and 2.88 Å. The aromatic ring centroid‐to‐centroid distance is 9.90 Å.

### Quantification of anion binding in silico

Computational methods were also used to explore the strength of anion binding by **1**. Binding energies were obtained by subtracting energies of optimised monomers from the total energy of the anion–transporter systems (for details, results and structures, see Experimental Section, Table 2 and Figure S13 in the Supporting Information, respectively). The calculations employed implicit DMSO solvation, so that the results could be related to the experimental measurements (see below). Note that all calculated energies of the optimised structures have inherent errors of approximately 1.0 kcal mol^−1^. Therefore, the binding energies in Table 2 provide a guide to which anions are strongly or weakly binding but cannot reliably predict the minor differences within both groups. To further assess the influence of transporter preorganization on anion binding, binding energies of the anions to a single arm of the transporter—modelled as methyl(*p*‐nitrophenyl)urea (see Supporting Information, Figure S14)—were calculated and are summarised in Table 2.

When comparing the binding energy of transporter **1** to twice the energy of a single ureido group, we obtained significant additional stabilisation energy (2.6–4.0 kcal mol^−1^) for **1⋅I^−^**, **1⋅Br^−^** and **1⋅H_2_PO_4_** ^**−**^. This suggests that decalin bis‐urea **1** might be well designed for binding these anions. Also, the complexation of NO_3_^−^, HSO_4_^−^ and Cl^−^ seems to be favoured by the binding site of **1**. In contrast, the binding energies calculated for **1⋅AcO^−^** and **1⋅HCO_3_** ^**−**^ are respectively 3.4 and 3.2 kcal mol^−1^ lower than those predicted from calculations on single arms, which might indicate that the binding geometry of **1** is not ideal for these types of anion.

### Affinities for anions in solution by NMR spectroscopy

**Figure 5:**
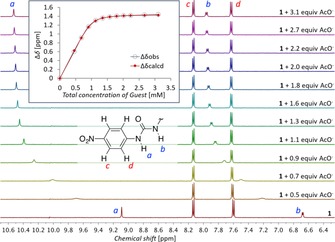
Downfield region of the ^1^H NMR spectra from the titration of receptor **1** (1 mm) with Bu_4_N^+^OAc^−^ in [D_6_]DMSO with 0.5 % H_2_O. The inset shows the observed binding curve for NH_a_ (blue) and the calculated fit for both NH signals (red).

To measure the binding constants of **1** in solution, ^1^H NMR titration experiments of **1** to anions were carried out in [D_6_]DMSO/H_2_O (200:1). The ^1^H NMR chemical‐shift changes of NH or CH (for H_2_PO_4_^−^ and SO_4_^2−^ the NH signal vanishes) were used to monitor the binding process (see Figure 5 for the titration with AcO^−^ as example), and the resulting binding constants *K*_a_ are listed in Table 2. With the exception of sulfate (see below), the binding curves could be fitted well to a 1:1 binding model (Figure 5 and Supporting Information). Affinities varied from <5 m ^−1^ for iodide to >10^4^ m ^−1^ for acetate, bicarbonate, dihydrogenphosphate and sulfate. Notably, the two regimes of binding constants obtained from ^1^H NMR titrations match the regimes found in the computational studies, with the *K*_a_ values <10^3^ m ^−1^ corresponding to binding energies lower than 17.7 kcal mol^−1^ and those >10^4^ m ^−1^ corresponding to more than 20 kcal mol^−1^.

**Figure 6:**
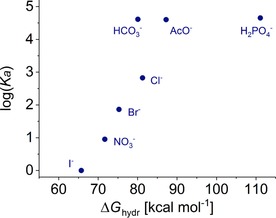
Correlation between the binding constants and the Gibbs free energies of hydration for anions considered in this work.

Given the wide range of affinities of **1** for different anions, it is tempting to conclude that the receptor shows impressive selectivity. However, the anions vary greatly in H‐bond acceptor strength (effectively “stickiness”). Indeed, the order of the affinity constants of **1** for the different anions correlates rather well with the Gibbs free energy of hydration of the anions (Table 2, Figure 6),^12^ an experimental indication of H‐bond acceptor ability. For example, both the hydration energy and our measured affinity constant for NO_3_^−^ are between the values for I^−^ and Br^−^. This implies that the variations in affinity depend more on the anion than the transporter. While transporter structure must have some influence, assessing this factor is not straightforward.^13^

To explore the effect of transporter structure, we employed an approach based on the analyses of Abraham^14^ and Hunter et al.,^15^, ^16^ in which the H‐bond donor and acceptor strengths are individually quantified through empirically derived parameters *α* and *β*. As shown by Hunter et al., the change in Gibbs free energy upon H‐bonding is expressed as Equation (1)

(1) $$\Delta G{}^{o}=-\left(\alpha -\alpha {}_{s}\right)\left(\beta -\beta {}_{s}\right)+6\;\mathrm{kJ}\;\mathrm{mol}{}^{-1}=-RT\;\ln\;K{}_{a}$$

in which *α* and *β* are parameters of H‐bond donor and acceptor, respectively, while *α*_s_ and *β*_s_ are corresponding parameters for the solvent.^16^ Relevant to anion recognition, *β* values for common anions have been measured and reported,^17^ and, as expected, roughly follow the hydration enthalpies shown in Figure 6.

Equation (1) is designed to describe the formation of a single H‐bond between a monovalent donor and acceptor, each of which is characterised by a single value of the pertinent *α* or *β* parameter. If applied to more complex systems, as in the present case, it can no longer be used predictively; geometric effects will perturb the binding strengths such that *α* and/or *β* will no longer be constant. However, if the equation is turned around so that measured affinities and standard values of one parameter (e.g., *β*) are used to calculate the other parameter (e.g., *α*), variations in the latter may be informative.

In the present case, we used the reported *β*_s_ and *α*_s_ values for DMSO,^16^ the reported *β* values for anions^17^ and the experimental *K*_a_ to calculate *α* for **1** when binding different anions. Some interesting variations are observed. For example, *α*=8 for **1⋅Cl^−^**, but 11 for **1⋅Br^−^**, and this suggests that **1** is better tailored to bind Br^−^ than Cl^−^. Note that whereas *K*_a_ for **1⋅Br^−^** in DMSO is only 70 m ^−1^, other binding studies on Br^−^ with urea‐ or thiourea‐based receptors in DMSO generally gave lower affinities,^18^, ^19^ unless Br^−^ was fully encapsulated in a cage.^20^ This is not to say that **1** is a poor receptor for Cl^−^; its affinity is still 3–35 times higher than those found for other urea‐based anion receptors with multiple (nitrophenyl)urea groups in DMSO.3e, 19, 21, 22, 23 However, it seems that the larger Br^−^ is a better fit than Cl^−^. This is also reflected in the unusual crystal structure of **1⋅Cl^−^** (Figure 4 a) as well as the similarly distorted calculated structure (Supporting Information, Figure S13) when compared to other anions studied.

Acetate is another case in which a relatively small value of *α*=6 for **1⋅AcO^−^** accompanies an apparently poor fit. This time the crystal structure suggests that the anion is too large for the binding site; the acetate anion adopts a twisted orientation relative to the urea units (Figure 3 d) and the inter‐urea distance is about 0.3 Å larger than in **1⋅**Br^−^. Again, **1** is nonetheless a good receptor for acetate when compared to others from the literature. The measured affinities are one to two orders of magnitude higher than those for comparable systems under the same conditions.^[3e19, 21, 22, 24]^

Considering tetrahedral anions, SO_4_^2−^ is very strongly bound by **1**, as often found for this substrate.3d Indeed the ^1^H NMR experiments indicate the binding of a second anion to form **1⋅(SO_4_** ^**2−**^ **)_2_**, despite the Coulomb repulsion between the two anions. Complex **1⋅HSO_4_** ^**−**^ is more weakly bound, as expected, but the *α* value of 13 suggests that the binding site is well‐adapted to this substrate. The affinity is higher than those previously observed for similar systems.3e, 19 H_2_PO_4_^−^ is also bound more strongly by **1** than by other compounds with the same binding motif.^19^, ^21^, ^22^, ^23^

Overall, the binding results confirm that **1** is especially well preorganised for anion recognition, showing high affinities for all anions measured. Positioning of the urea groups on the decalin core ensures an appropriate distance and binding angle between the urea groups, while the restricted rotation about the axial C−N bonds prevents intramolecular H‐bond formation.^5^, ^8^ The arms show significant flexibility (also rotational), permitting the intramolecular distances between the upper and the lower urea nitrogen atoms to range between 4.9–5.4 and 5.5–7.9 Å, respectively. This allows binding of a broad range of anions with high affinities. On the other hand, the rigid decalin framework does affect selectivity, favouring (for example) bromide over chloride and acetate.

## Conclusion

We have shown that diureidodecalin **1**, a non‐toxic anionophore of exceptional interest for biological applications, is an effective receptor for a range of anions. The complexes were structurally characterised and investigated by crystallographic and computational techniques. The results highlight the preorganisation conferred by the *trans*‐1,5‐diureidodecalin framework, which prevents intramolecular H‐bonding between the two arms and promotes high anion affinities. At the same time, the flexibility of the urea units allows binding of various anions of different geometries. Binding constants are largely determined by the physicochemical properties of the anion, but analysis in terms of the H‐bonding parameters *α* and *β* provides a means of assessing the effects of receptor structure. Notably, the anions which seem to be disfavoured according to this analysis (chloride and acetate) are also those for which the complexes show unusual structural features. The in‐depth characterisation of the binding properties of **1** will help provide a platform for biological applications of the promising diureidodecalin family of anion transporters.

## Experimental Section

### Crystallisation and X‐ray analysis

Complexes **1⋅X^−^** were crystallised with Me_4_N^+^, Et_4_N^+^ or Bu_4_N^+^ countercations from solvents or solvent mixtures by slow evaporation at room temperature. Compound **1** was also crystallised by itself in various solvate crystal structures. Some other solvates were fortuitously obtained during crystallisation with anion salts. Detailed conditions under which the different crystals were obtained are reported in the Supporting Information.

Data for **1⋅acetone**, **1⋅H_2_O**, **1⋅THF** and **1⋅SO_4_** ^**2−**^ were collected at 123(2) K with a dual‐source Rigaku SuperNova Oxford diffractometer equipped with an Atlas detector by using mirror‐monochromated Cu_Kα_ radiation (*λ*=1.54184 Å). Single‐crystal X‐ray data for **1⋅AcO^−^** and **1⋅NO_3_** ^**−**^ were collected at 120(2) K with a Rigaku SuperNova Oxford single‐source diffractometer fitted with an Atlas EoS CCD detector by using mirror‐monochromated Mo_Kα_ (*λ*=0.71073 Å) radiation. X‐ray diffraction experiments on **1⋅MeOH**, **1⋅Cl^−^** and **1⋅Br^−^** were carried out at 100(2) K with a Bruker APEX II CCD diffractometer by using Mo_Kα_ radiation (*λ*=0.71073 Å). The data collection and reduction carried out with Rigaku SuperNova Oxford diffractometers were performed with the program CrysAlisPro,^25^ and the data for **1⋅DMSO** obtained with the Bruker Nonius Kappa diffractometer were processed with the programs COLLECT,^26^ HKL DENZO and SCALEPACK.^27^ The intensities were corrected for absorption by using the Gaussian face‐index absorption correction method^25^ for **1⋅acetone**, **1⋅H_2_O**, **1⋅THF**, **1⋅DMSO**, **(1)_2_⋅SO_4_** ^**2−**^, **1⋅AcO^−^** and **1⋅NO_3_** ^**−**^, and the intensities for **1⋅MeOH**, **1⋅Cl^−^** and **1⋅Br^−^** were integrated in SAINT and absorption corrections were based on equivalent reflections by using SADABS^28^ with multiscan absorption correction method. The structure of **1⋅Cl^−^** was solved with Superflip.^29^ Structures of **1⋅MeOH**, **1⋅acetone**, **1⋅THF**, **(1)_2_⋅SO_4_** ^**2−**^, **1⋅AcO^−^**, **1⋅Br^−^** and **1⋅NO_3_** ^**−**^ were solved with direct methods (SHELXS)^30^ and refined by full‐matrix least‐squares techniques on *F* ^2^ in OLEX2,^31^ by using SHELXL.^30^ Constraints and restraints were used where appropriate for disordered models.

Crystal structure and refinement data are given in Tables S1 and S2 in the Supporting Information. https://summary.ccdc.cam.ac.uk/structure-summary?doi=10.1002/chem.201800537 1818063 (**1⋅H_2_O**), 1817830 (**1⋅acetone**), 1817831 (**1⋅THF**), 1586249 (**1⋅1.36 CH_3_OH⋅0.64 H_2_O**), 1817832 (**1⋅DMSO**), 1817834 (**1⋅NO_3_** ^**−**^), 1586251 (**1⋅Br^−^**), 1817833 (**1⋅AcO^−^**) and 1817835 [**(1)_2_⋅SO_4_** ^**2−**^] contain the supplementary crystallographic data for this paper. These data can be obtained free of charge from http://www.ccdc.cam.ac.uk/.

### Computational studies

Optimizations were performed with the program TURBOMOLE version 7.00^32^ by using DFT (TPSS^33^/Def2‐TZVPPD).^34^ Further acceleration of calculations was achieved by using the MARI‐J approach.^35^ Empirical Grimme's dispersion correction^36^ with Becke–Johnson damping^37^ (D3‐BJ) was employed. DMSO (COSMO) implicit solvation^38^ was used to mimic the solution environment. The starting geometries were typically taken from the crystal structures and slightly modified if necessary.

### Binding studies in solution

Guests were obtained commercially as *n*Bu_4_N^+^ salts, except for the bicarbonate salt Et_4_NHCO_3_ and sulfate, which was purchased as a 50 wt % solution of (Bu_4_N)_2_SO_4_ in H_2_O and lyophilised to obtain the solid. Transporter **1** was synthesized as reported previously.^4^ Binding constants of complexes **1⋅X^−^** were obtained by ^1^H NMR titration experiments with 0.5 % H_2_O in [D_6_]DMSO. The binding constant for **1⋅**Cl^**−**^ with Bu_4_NCl was previously reported.^4^

Compound **1** was dissolved in [D_6_]DMSO with 0.5 % H_2_O at 1.0 mm and for each titration 500 μL of this solution was placed in an NMR tube. The guests were dissolved in the solution of **1** (1.0 mm) in [D_6_]DMSO with 0.5 % H_2_O, so that the concentration of host did not decrease over the course of the experiment. Concentrations of the guests and the volumes of the aliquots of guest solution added to the host solution during the titration were varied over the experiments, depending on the strength of the observed interaction. All ^1^H NMR titration binding studies were performed with a Varian 500 MHz NMR spectrometer with a proton sensitive probe at 298 K.

The ^1^H NMR chemical‐shift changes of NH or CH groups were used to monitor the binding process. Binding constants were determined by fitting the shifts of multiple resonances (see Supporting Information) to a 1:1 binding model with a least‐squares fitting procedure in a custom‐made Excel spreadsheet. The errors reported in the Supporting Information represent the standard deviation of the binding constants calculated for individual measured points. The data obtained for the titration with sulfate could not be fitted to a 1:1 binding model, and therefore the online fitting application at http://supramolecular.org/ was used to fit the data to a 1:2 binding model.

## Conflict of interest

The authors declare no conflict of interest.

## Supporting information

As a service to our authors and readers, this journal provides supporting information supplied by the authors. Such materials are peer reviewed and may be re‐organized for online delivery, but are not copy‐edited or typeset. Technical support issues arising from supporting information (other than missing files) should be addressed to the authors.

Supplementary
