# Supplementary material for: Anion Recognition by a Bioactive Diureidodecalin Anionophore: Solid‐State, Solution, and Computational Studies
Source: Chemistry. 2018 May 14;24(32):8178–85. doi: 10.1002/chem.201800537 (PMC6055605; doi:10.1002/chem.201800537)
Supplement: Supplementary file 1 — Supplementary [file CHEM-24-8178-s001.pdf]

CHEMISTRY
A European Journal

Supporting Information

Anion Recognition by a Bioactive Diureidodecalin Anionophore: Solid-State, Solution, and Computational Studies

Ondřej Jurček+,[a, b, c] Hennie Valkenier+,[b, d] Rakesh Puttreddy,[a] Martin Novák,[c] Hazel A. Sparkes,[b] Radek Marek,*[c, e] Kari Rissanen,*[a] and Anthony P. Davis*[b]

chem_201800537_sm_miscellaneous_information.pdf

Supporting Information

Contents

1 X-ray crystallography S2

2 Computational studies S12

3 NMR titrations with various anions in DMSO with 0.5% H_2O S15

4 References S22

1 X-ray crystallography

X-ray diffraction experiments on **1·MeOH**, **1·Cl**$^-$, and **1·Br**$^-$ were carried out at 100(2) K on a Bruker APEX II CCD diffractometer using Mo-K$_\alpha$ radiation (λ = 0.71073 Å). Intensities were integrated in SAINT[1] and absorption corrections were based on equivalent reflections using SADABS.[2] Structures **1·MeOH** and **1·Br**$^-$ were solved using olex2.solve[3] while **1·Cl**$^-$ was solved using Superflip[4, 5]. All of the structures were refined against F^2 in SHELXL[6,7] using Olex2[3]. All of the non-hydrogen atoms were refined anisotropically. While all of the hydrogen atoms were located geometrically and refined using a riding model. In the case of **1·MeOH** the molecule displayed disorder, the occupancies of the fragments were determined by refining them against a free variable with the sum of the two sites set to equal 1, the occupancies were then fixed at the refined values. Restraint and constraints were placed on some of the thermal and geometric parameters to maintain sensible values. Solvent in part of the lattice was modelled as either two partially occupied water molecules or a methanol with refined relative occupancies 64:36. In **1·Br**$^-$ the central pair of rings in **1** were modelled as disordered over two positions. The occupancies were refined with the sum set to equal 1 and subsequently fixed at the refined values of 65:35, restraints and constraints were applied to maintain sensible thermal and geometric parameters. The NMe_4^+ counterion was treated in the same manner. Data for **1·acetone**, **1·H$_2$O**, **1·THF** and **1·SO$_4^{2-}$** were measured using a dual source Rigaku SuperNova Oxford diffractometer equipped with an Atlas detector using mirror-monochromated Cu-Kα radiation (λ = 1.54184 Å). Single crystal X-ray data for **1·AcO**$^-$ and **1·NO$_3$**$^-$ were collected using Rigaku SuperNova Oxford single-source diffractometer suited with Atlas EoS CCD detector using mirror-monochromated Mo-Kα (λ = 0.71073 Å) radiation. The data collection for **1·DMSO** was performed using Bruker-Nonius Kappa CCD diffractometer with an APEX-II detector and graphite mono-chromatised Mo-Kα (λ = 0.71073 Å) radiation. The data collection and reduction carried out on Rigaku SuperNova Oxford diffractometers were done using the program CrysAlisPro,[8] and the data obtained from Bruker Nonius Kappa diffractometer were processed using the program COLLECT[9] and HKL DENZO AND SCALEPACK[10]. The intensities were corrected for absorption using either Analytical or Gaussian face-index absorption correction method[8] for **1·acetone**, **1·H$_2$O**, **1·THF**, **1·SO$_4^{2-}$**, **1·AcO**$^-$ and **1·NO$_3$**$^-$, and the intensities for **1·DMSO** were corrected for absorption using SADABS[6,7] with multi-scan absorption correction type method. All the structures were solved with direct methods (SHELXS)[9] and refined by full-matrix least squares on F^2 using the OLEX2[7], which utilizes the SHELXL-2014 module.[9] No attempt was made to locate the hydrogens for disordered solvent molecules. Constraints and restraints are used where appropriate for disordered models.

Crystal structure and refinement data are given in Table S1 and S2. Crystallographic data for all compounds have been deposited with the Cambridge Crystallographic Data Centre as supplementary publication CCDC ####-####. Copies of the data can be obtained free of charge on application to CCDC, 12 Union Road, Cambridge CB2 1EZ, UK [fax (+44) 1223 336033, e-mail: deposit@ccdc.cam.ac.uk].

Acetone solvate, **1·Acetone**: **1** was dissolved in acetone and the solution was slowly evaporated to obtain X-ray quality single crystals. Interestingly, when crystals of acetone solvate are placed in the atmosphere of THF vapours THF solvate is obtained (replacement of solvent molecules among zig-zag layers, "breathing crystals", see also solvate of tetrahydrofuran below).

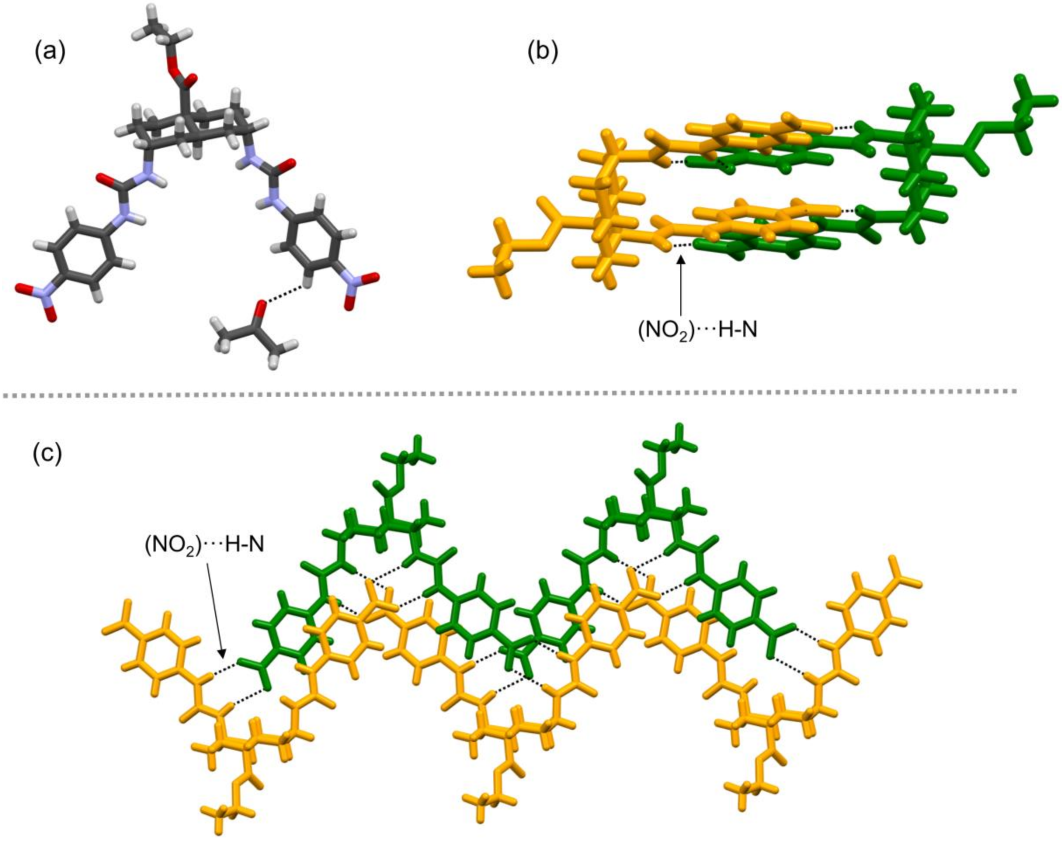

Figure S1. Asymmetric unit of **1·Acetone** in stick model. Representation: Golden yellow and green sticks are receptors. Black broken lines represent hydrogen bonds.

Hydrate, **1·H_2O**: **1** was dissolved in dimethyl sulfoxide and methanol solution of TMAOH was added in excess. The mixture was evaporated on a watch glass to yield X-ray quality single crystals.

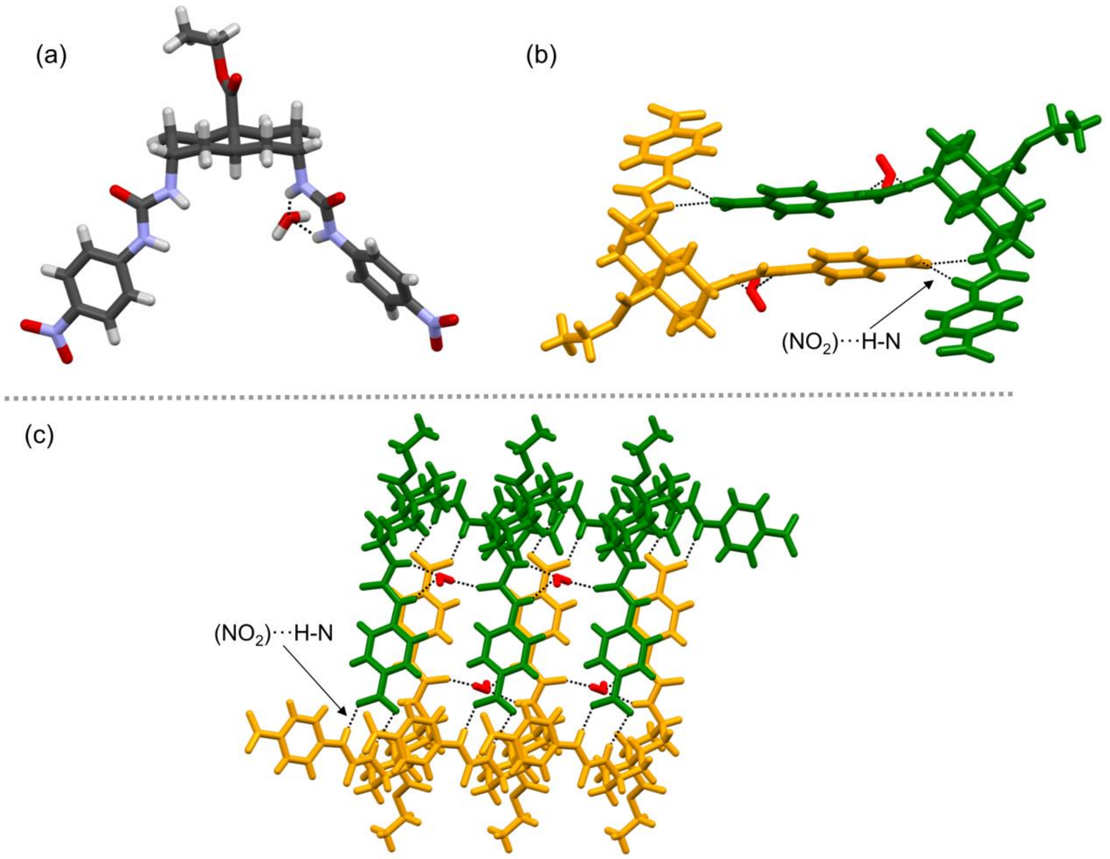

Figure S2. (a) Asymmetric unit of **1·H_2O** in stick model. Side- (b) and top-view (c) of 1-D polymers to display hydrogen bond interaction of nitro and urea group of adjacent transporter **1**. Representation: Golden yellow and green sticks are receptor, and red are water molecules. Black broken lines represent hydrogen bonds.

Tetrahydrofuran solvate, **1·THF**: **1** was dispersed in tetrahydrofuran and acetone was added until the solid was fully dissolved. The mixture was slowly evaporated to obtain X-ray quality single crystals which were analysed. Crystallization has led to potentially porous structure, where four molecules of tetrahydrofuran template the channel.

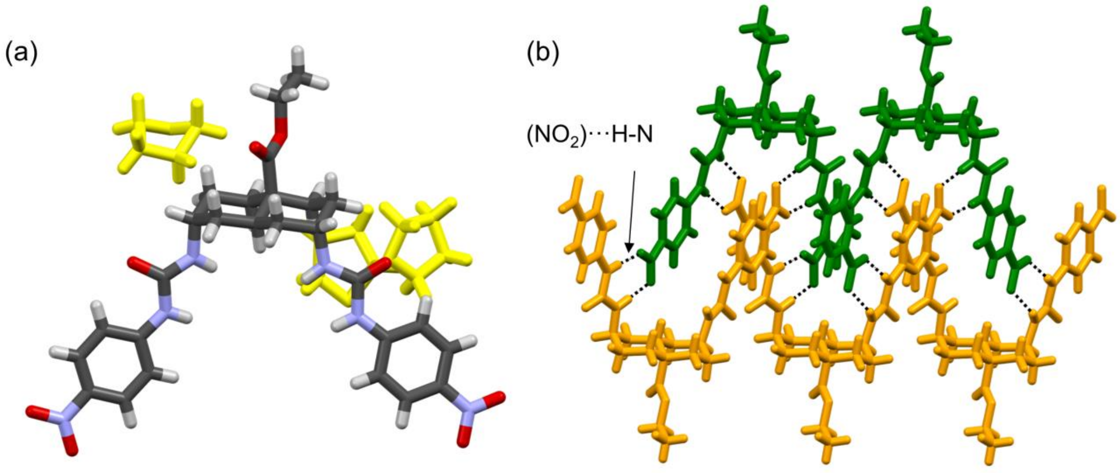

Figure S3. (a) Asymmetric unit of **1·THF** in stick model. Solvent molecules are shown in yellow sticks. (b) Golden yellow and green sticks are receptors. Disordered components are omitted for viewing clarity. Black broken lines represent hydrogen bonds.

Dimethyl sulfoxide solvate, **1·DMSO**: **1** was dissolved in dimethyl sulfoxide, the vial was closed and left standing in the fume-hood. X-ray quality single crystals have grown on the solvent surface after one week.

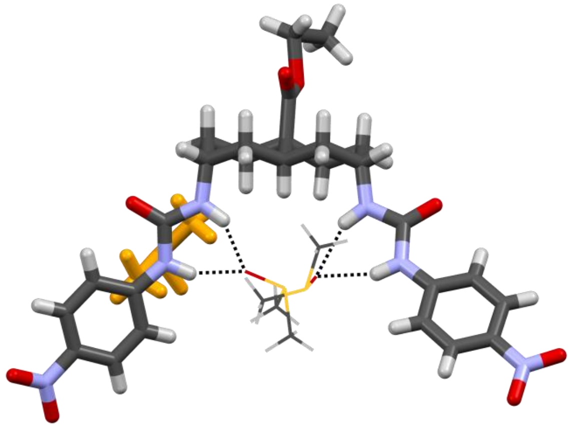

Figure S4. Asymmetric unit of **1·DMSO** in stick model. Selected solvent molecules are shown in golden yellow sticks and wireframe model for viewing clarity. Disordered components are omitted for viewing clarity. Black broken lines represent hydrogen bonding.

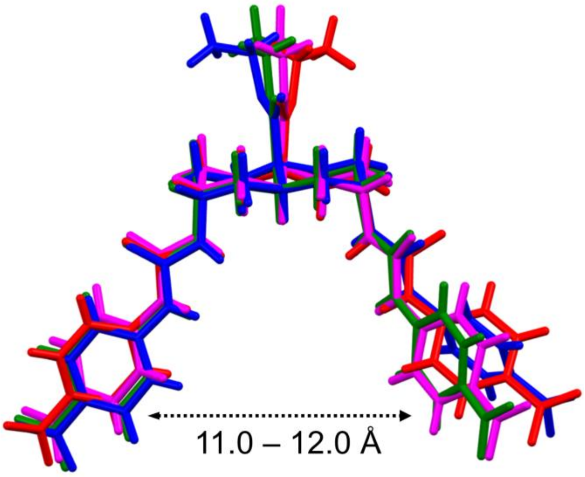

Figure S5. Overlay of receptors **1** crystallised in “open” type conformations from different solvents: The solvents and disordered components were omitted for clarity. Representation: **1·H$_2$O** (blue), **1·Acetone** (green), **1·THF** (magenta), and **1·DMSO** (red).

Methanol solvate, **1·MeOH**: **1** was dissolved in the mixture of methanol with a small amount of water (5 %) and the mixture was slowly evaporated yielding X-ray quality single crystals.

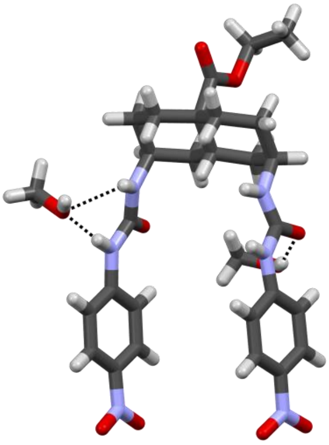

Figure S6. Asymmetric unit of **1·MeOH** in stick model. Black broken lines represent hydrogen bonding.

Table S1. Crystal data and X-Ray experimental details for solvates.

	**1·H_2O**	**1·Acetone**	**1·THF**	**1·DMSO**	**1.$(CH_3OH)_{1.36}(H_2O)_{0.64}$**
**CCDC No.**	1818063	1817830	1817831	1817832	1586249
**Chemical formula**	$C_{27}H_{34}N_6O_9$	$C_{30}H_{38}N_6O_9$	$C_{31}H_{40}N_6O_9$	$C_{33}H_{50}N_6O_{11}S_3$	$C_{28.36}H_{39.69}N_6O_{10.64}$
M_r	586.60	626.66	640.69	802.97	634.91
**Temperature (K)**	120.01(10)	123(2)	123(2)	170(2)	100(2)
**Crystal system, space group**	Trigonal *R*-3	Triclinic *P*-1	Triclinic *P*-1	Triclinic *P*-1	Triclnic *P*-1
***a*, *b*, *c* (Å)**	46.5005(10) 46.5005(10) 6.79920(16)	7.0007(7) 14.5563(14) 15.2431(12)	8.9631(3) 11.6680(5) 19.3211(7)	12.421(3) 13.419(3) 14.700(3)	6.5543(2) 13.7341(3) 18.2906(5)
**α, β, γ (°)**	90 90 120	88.980(7) 87.912(7) 89.368(8)	77.807(3) 84.628(3) 80.193(3)	100.87(3) 103.69(3) 117.12(3)	107.616(1) 91.119(2) 98.590(1)
**Volume (Å^3)**	12732.2(6)	1552.0(2)	1942.68(12)	1990.3(9)	1547.99(7)
Z	18	2	2	2	2
**Density (Calculated) mg/m^3**	1.377	1.341	1.095	1.340	1.362
**Absorption Coefficient (mm^{-1})**	0.880	0.837	0.678	0.249	0.105
**F(000)**	5580	664	680	852	674
**Crystal size (mm^3)**	0.21 x 0.07 x 0.04	0.14 x 0.05 x 0.04	0.19 x 0.12 x 0.08	0.17 x 0.15 x 0.12	0.43 x 0.34 x 0.18
**θ range for data collection (°)**	3.29 to 66.75	4.16 to 65.73	3.92 to 66.75	1.82 to 27.50	1.171 to 26.777
**Reflections collected [R(int)]**	13576 [0.0931]	9176 [0.1045]	11382 [0.0396]	18850 [0.0540]	25427 [0.0339]
**Reflections [I>2sigma(I)]**	3316	2847	4877	3796	4985
**Data completeness (%)**	97.90	99.01	99.02	98.90	99.70
**Data/ restraints/ parameters**	4906/6/390	5455/0/409	6818/115/603	9030/0/493	6602/171/481
**Goodness-of-fit on F^2**	1.033	1.046	1.275	1.026	1.033
**Final R_1 indices [I>2sigma(I)]**	R_1 = 0.0686 wR_2 = 0.1691	R_1 = 0.0727 wR_2 = 0.1688	R_1 = 0.1167 wR_2 = 0.3091	R_1 = 0.1029 wR_2 = 0.2303	R_1 = 0.0433 wR_2 = 0.1034
**Final R indices [all data]**	R_1 = 0.1096 wR_2 = 0.2497	R_1 = 0.1336 wR_2 = 0.2254	R_1 = 0.1410 wR_2 = 0.3431	R_1 = 0.2283 wR_2 = 0.2938	R_1 = 0.0631, wR_2 = 0.1134
**Largest diff. peak/hole (e.Å^{-3})**	0.653/ -0.685	0.233/ -0.255	1.205/ -0.433	0.929/ -0.452	0.30/ -0.25

Receptor 1 ⊃ TMA salts

1 ⊃ TMACl, **1·Cl⁻**: **1** (1 mg) was submerged in chloroform (slightly soluble) and concentrated methanol solution of TMACl (1.1 eq.) was added after which **1** got fully dissolved. Subsequently, within the first minute thin fibres were formed throughout the whole volume of the mixture. At this point, the vial was closed and left standing in the fume-hood. Within one hour of the experiment fibrous material transformed into X-ray quality single crystals, which were successfully analysed.

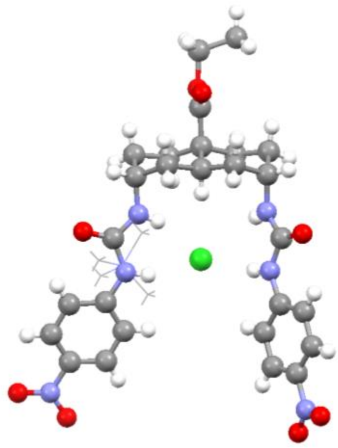

Figure S7. X-ray crystal structure of **1** with TMACl (receptor **1** and chloride are presented in ball and stick model, TMA^+ is in wireframe model).

1 ⊃ TMABr, **1·Br⁻**: **1** (1 mg) was submerged in chloroform (slightly soluble) and concentrated methanol solution of TMABr (1.1 eq.) was added after which **1** got fully dissolved. The mixture was slowly evaporated to obtain X-ray quality single crystals in two days.

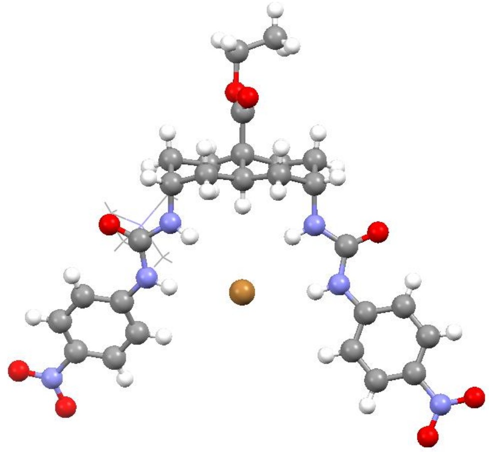

Figure S8. X-ray crystal structure of **1** with TMABr (receptor **1** and bromide are presented in ball and stick model, TMA^+ is in wireframe model).[1]

[1] In **1·Br⁻** the central pair of rings in **1** were modelled as disordered over two positions. The occupancies were refined with the sum set to equal 1 and subsequently fixed at the refined values of 65:35, restraints and constraints were applied to maintain sensible thermal and geometric parameters. The Me_4N^+ counterion was treated in the same manner.

1 ⊃ $TMANO_3$, **1·NO_3^-**: **1** (1 mg) was submerged in tetrahydrofuran (insoluble) and strong methanol solution of $TMANO_3$ (1.1 eq.) was added after which **1** got fully dissolved. The solution was slowly evaporated to obtain X-ray quality single crystals in three days.

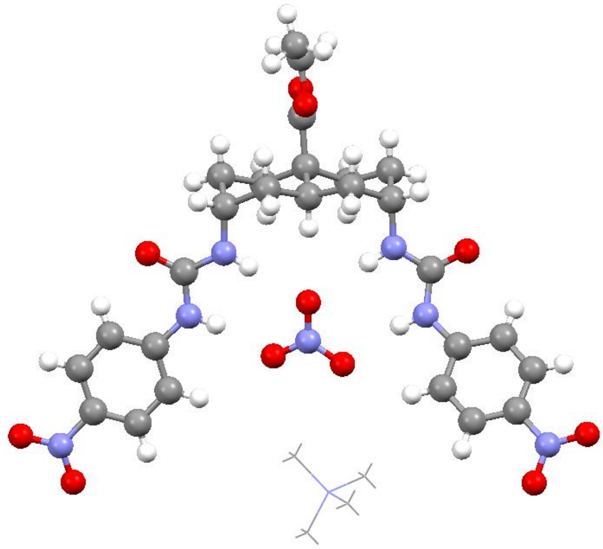

Figure S9. X-ray crystal structure of **1** with $TMANO_3$ (receptor **1** and nitrate are presented in ball and stick model, TMA^+ is in wireframe model).

1 ⊃ TMA(AcO), **1·AcO^-**: TMA(AcO) salt was initially prepared by mixing acetic acid with methanol solution of TMAOH in ratio 1:1. **1** (1 mg) was submerged in ethanol (very slightly soluble) and methanol solution of TMA(AcO) (1.1 eq.) was added after which **1** got fully dissolved. The mixture was slowly evaporated to give X-ray quality single crystals.

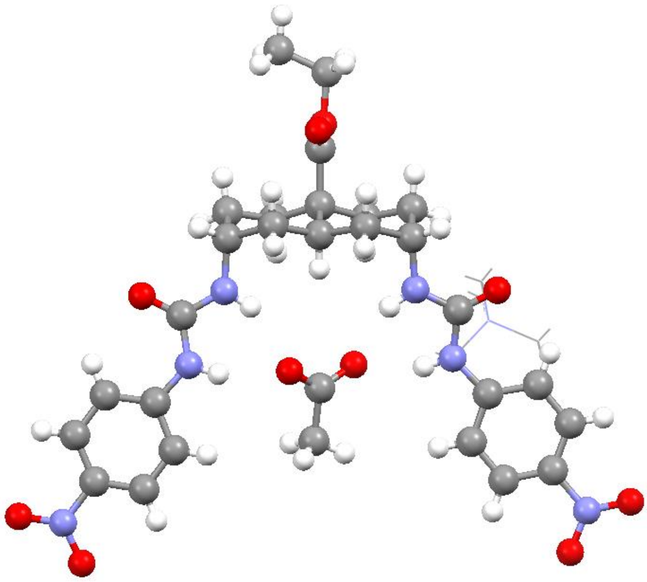

Figure S10. X-ray crystal structure of **1** with TMA(AcO) (receptor **1** and acetate are presented in ball and stick model, TMA^+ is in wireframe model).

1 ⊃ $(TMA)_2SO_4$, **1·(SO_4^{-2})$_2$**: **1** (1 mg) was submerged in ethanol (very slightly soluble) and concentrated methanol solution of $(TMA)_2SO_4$ (1.1 eq.) was added after which **1** got mostly dissolved. The solution was sonicated, heated, filtered and finally slowly evaporated to obtain X-ray quality single crystals.

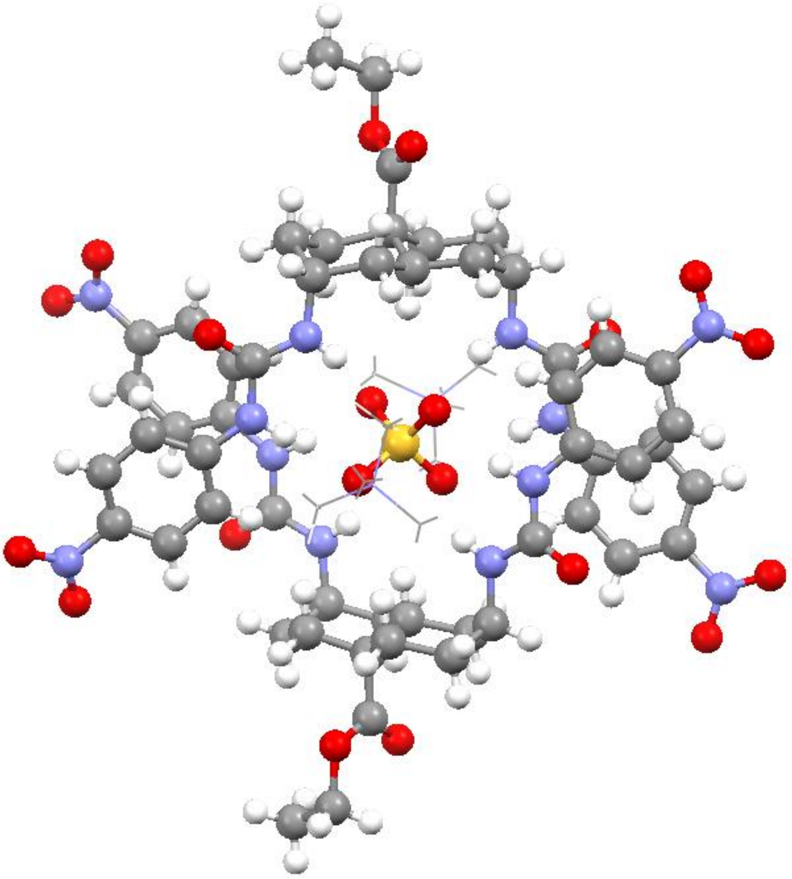

a)

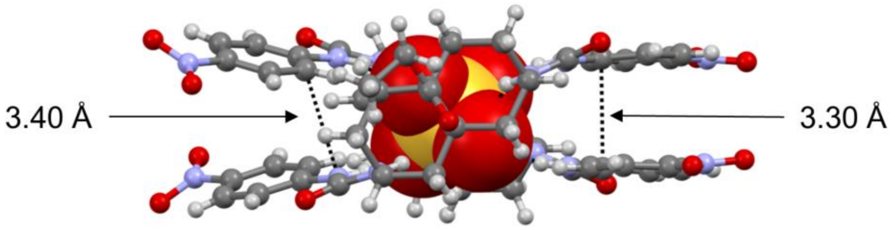

b)

Figure S11. a) X-ray crystal structure of **1** with $(TMA)_2SO_4$ (receptor **1** and sulfate are presented in ball and stick model, TMA^+ is in wireframe model), b) side view of dimer to show weak π-π contacts. The receptor **1** is shown in ball and stick model and sulphate in CPK model. Black broken lines represent short contacts between aromatic rings.

Table S2. Crystal data and X-ray experimental details for **1**·anion complexes.

	**1·NO_3^-**	**1·Br^-**	**1·Cl^- lit.11**	**1·AcO^-**	**1·SO_4^{2-}**
**CCDC No.**	1817834	1586251	1420195	1817833	1817835
**Chemical formula**	$C_{31}H_{44}N_8O_{11}$	$C_{31}H_{44}BrN_7O_8$	$C_{31}H_{44}ClN_7O_8$	$C_{66}H_{96}N_{14}O_{21}$	$C_{62}H_{92}N_{14}O_{22}S$
***M*r**	704.74	722.64	678.18	1421.56	1417.55
**Temperature (K)**	120.0(1)	100(2)	100(2)	120.0(1)	123.01(10)
**Crystal system, space group**	Triclinic *P*-1	Triclinic *P*-1	Monoclinic *P*2$_1$/*n*	Triclinic *P*-1	Orthorhombic *Fddd*
***a*, *b*, *c* (Å)**	9.7778(5) 12.4706(6) 15.6404(7)	9.3206(3) 12.4296(4) 16.1619(4)	12.1881(3) 24.5629(6) 12.5908(3)	10.6379(7) 11.5474(7) 16.0836(7)	24.2871(15) 25.8633(8) 44.6306(17)
**α, β, γ (°)**	80.496(4) 71.891(4) 69.465(5)	84.575(2) 74.297(2) 68.650(2)	90 117.009(2) 90	78.225(4) 88.215(4) 65.449(6)	90 90 90
**Volume (Å^3)**	1694.09(15)	1678.78(9)	3358.3(2)	1756.09(19)	28034(2)
Z	2	2	4	1	16
**Density (Calculated) mg/m^3**	1.423	1.430	1.341	1.344	1.343
**Absorption Coefficient (mm^{-1})**	0.106	1.284	0.174	0.101	1.125
**F(000)**	748	756	1440	758	12064
**Crystal size (mm^3)**	0.15 x 0.08 x 0.04	0.38 x 0.39 x 0.11	0.36 x 0.24 x 0.20	0.18 x 0.09 x 0.07	0.26 x 0.12 x 0.06
**θ range for data collection (°)**	3.00 to 25.25	1.31 to 26.98 2.618 to 53.952	1.66 to 26.78 3.316 to 53.554	3.18 to 27.50	3.88 to 66.75
**Reflections collected [R(int)]**	11937 [0.0517]	27157 [0.0563]	27306 [0.0517]	14523 [0.0306]	40123 [0.0462]
**Reflections [I>2sigma(I)]**	3581	5176	5155	5769	4687
**Data completeness (%)**	99.75	99.30	99.70	99.83	99.97
**Data/ restraints/ parameters**	6113 /36/572	7254 /316/584	7158/2/435	8032/0/566	6217/48/452
**Goodness-of-fit on F^2**	1.043	1.022	1.019	1.078	1.895
**Final R_1 indices [I>2sigma(I)]**	R_1 = 0.0761 wR_2 = 0.1720	R_1 = 0.0551 wR_2 = 0.1092	R_1 = 0.0433 wR_2 = 0.0889	R_1 = 0.0714 wR_2 = 0.1597	R_1 = 0.1495 wR_2 = 0.4462
**Final R indices [all data]**	R_1 = 0.1356 wR_2 = 0.2098	R_1 = 0.0885 wR_2 = 0.1224	R_1 = 0.0706 wR_2 = 0.0994	R_1 = 0.1008 wR_2 = 0.1744	R_1 = 0.1629 wR_2 = 0.4666
**Largest diff. peak/hole (e.Å^{-3})**	0.712/ -0.512	0.62 /-0.60	0.65/-0.22	0.395/-0.357	1.036/-0.653

2 Computational studies

Two conformations corresponding to stationary points on the potential energy surface were used as references for binding energy calculations: a) with the arms perpendicular to each other (Fig. S27 a), and b) with coplanar arms (Fig. S27 b). Conformation with coplanar arms resulted in lower total energy, however, additional stabilization with DMSO molecule bound in place of the anion might result in further stabilization (rotation can be an artefact of the continuum model). This suggestion comes from the observation in the crystal structures of DMSO solvate, where two molecules of DMSO are H-bonded to the ureas (Fig. S18).

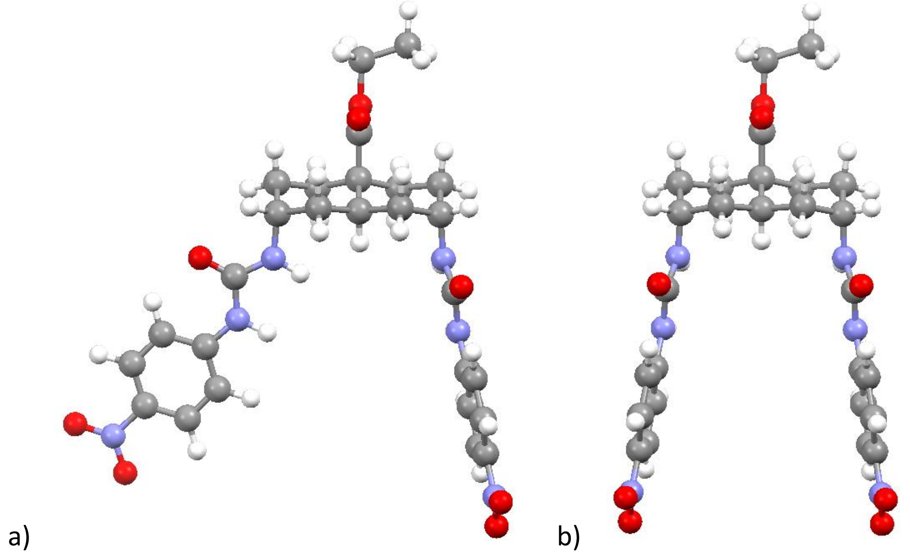

Figure S12. Two conformations corresponding to reference states: a) arms perpendicular to each other, and b) coplanar arms.

The crystal structure of the bromide complex **1·Br**$^{-}$ was used as a template for computational modelling of the complexes with spherical anions, while the nitrate crystal structure was used for modelling of trigonal-planar complexes, and the sulfate crystal structure was used for the tetrahedral complexes. In the nitrate crystal structure, the receptor ethyl ester adopted a different orientation from those in all other systems. It has been found that this conformational difference increases the total energy of the complex, thus the ethyl ester group was adjusted manually to maintain a consistent geometry of **1** throughout the whole series.

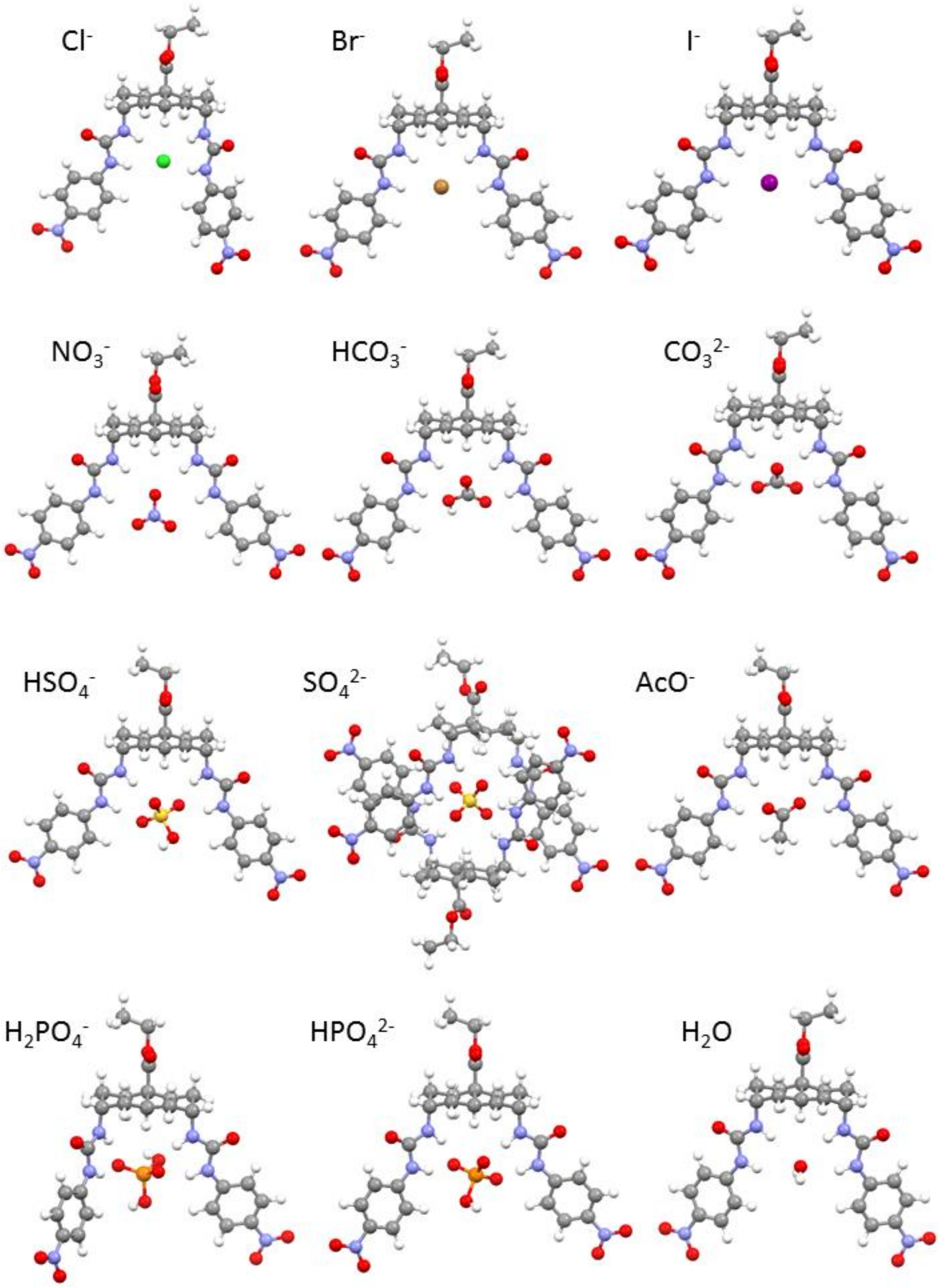

Figure S13. DFT optimized structures of complexes of **1** with various anions and water in DMSO implicit solvent.

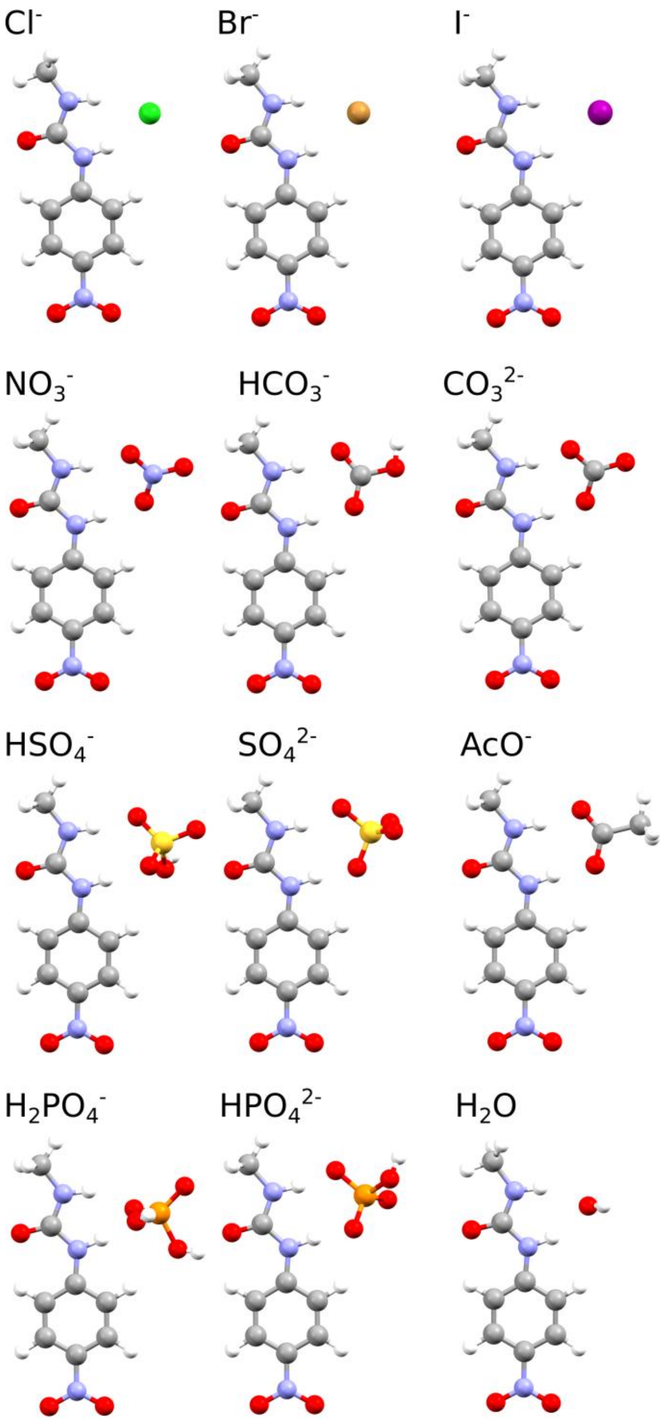

Figure S14. Optimized structures of methyl(*p*-nitrophenyl)urea (single arm) with various anions and water in DMSO implicit solvent.

3 NMR titrations with various anions in DMSO with 0.5% H_2O

^1H NMR titration of 1 with $Bu_4N^+Br^-$ in DMSO-d6/H_2O (200:1)

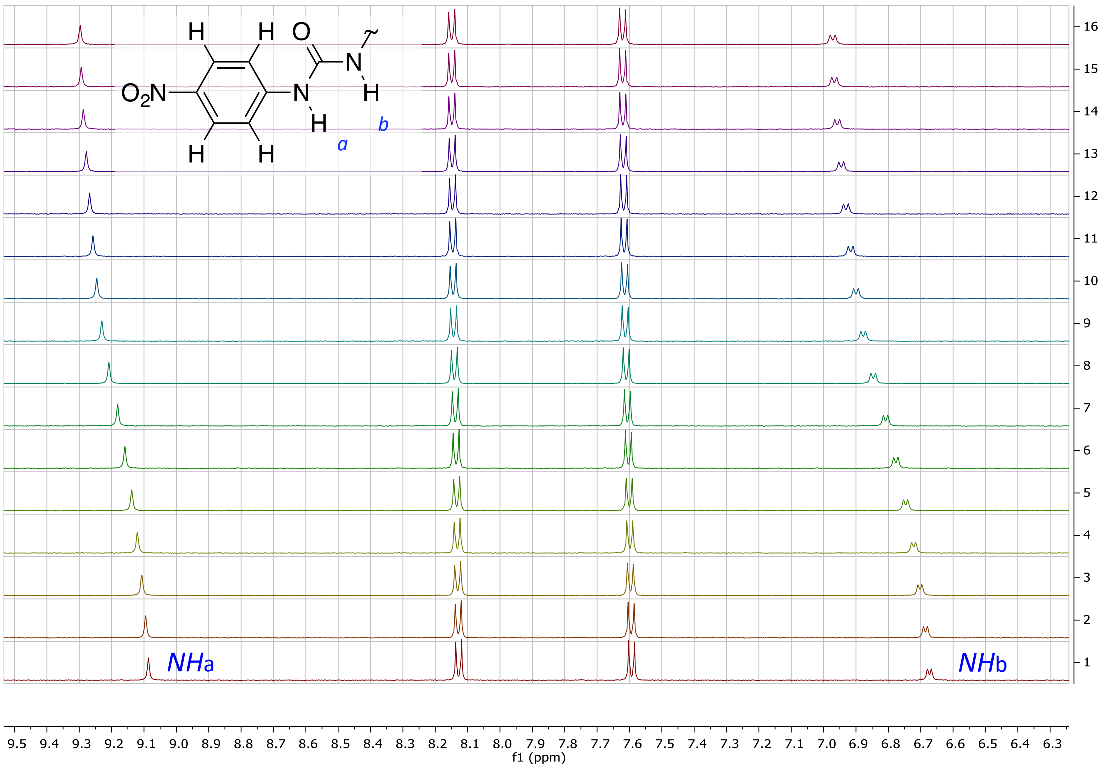

Figure S15. Downfield region of the ^1H NMR spectra from the titration of $Bu_4N^+Br^-$ into 1 mM **1** in DMSO with 0.5% H_2O.

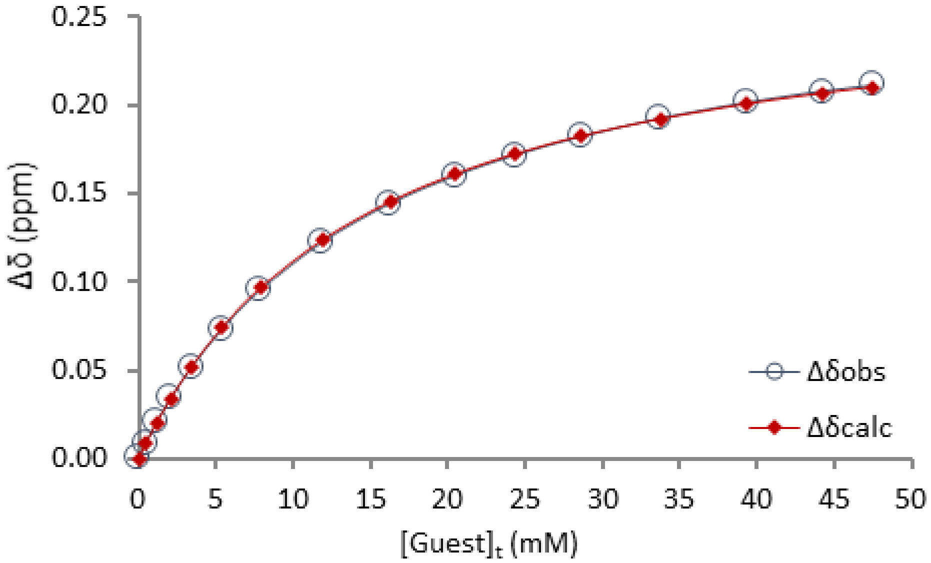

Figure S16. Graph showing the observed binding curve (for NH_a) and calculated fitting for **1** when titrated against $Bu_4N^+Br^-$ at 298 K. The movements of both NH signals were used to find $K_a = 73 \pm 1\ M^{-1}$.

^{1}H NMR titration of 1 with $Bu_4N^+I^-$ in DMSO-d6/H_2O (200:1)

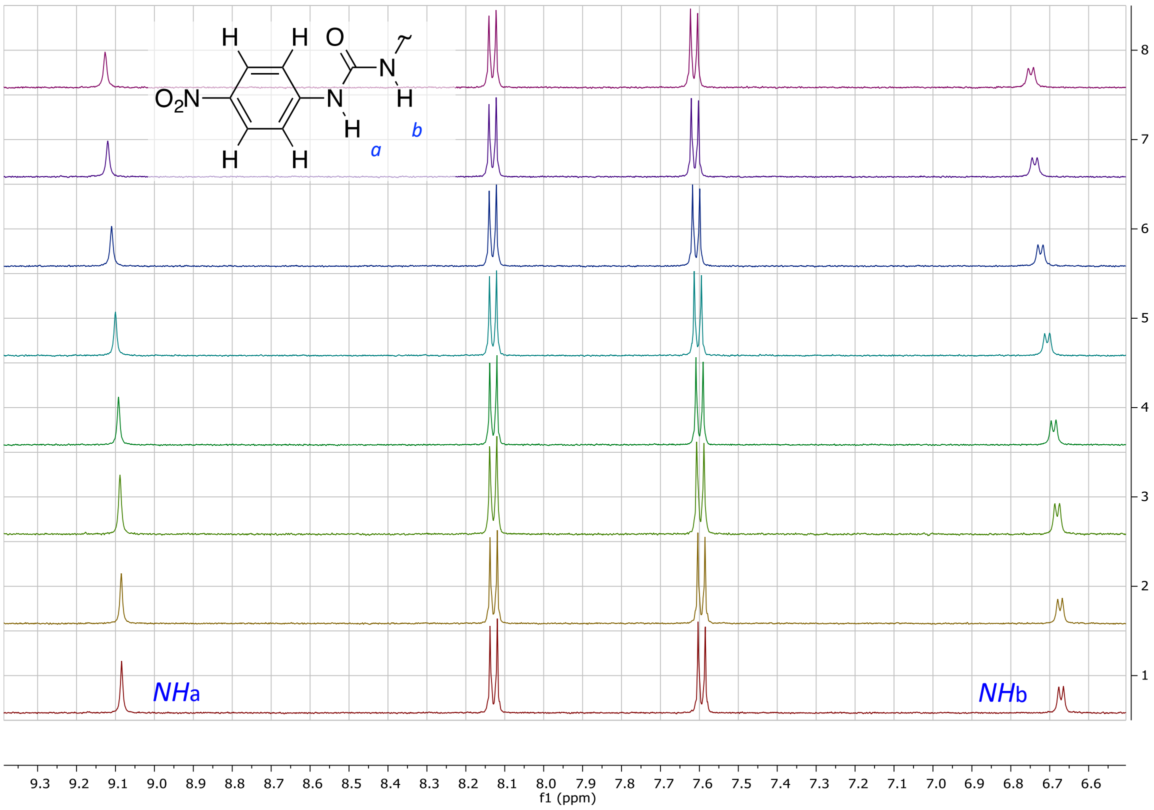

Figure S17. Downfield region of the ^{1}H NMR spectra from the titration of $Bu_4N^+I^-$ into 1 mM **1** in DMSO with 0.5% H_2O.

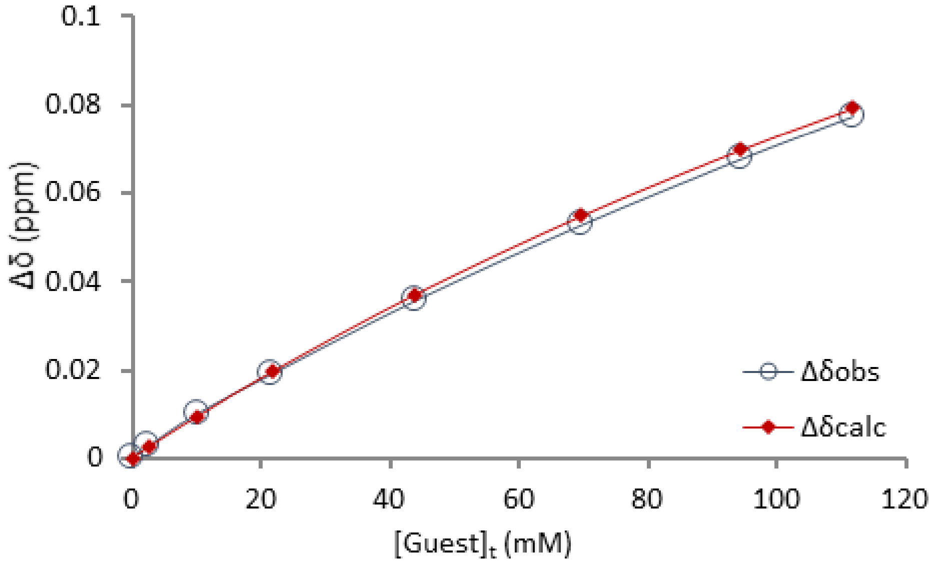

Figure S18. Graph showing the observed binding curve (for NH_b) and calculated fitting for **1** when titrated against $Bu_4N^+I^-$ at 298 K. The movements of both NH signals were used to find K_a ~ 3 M^{-1}.

^1H NMR titration of 1 with $Bu_4N^+NO_3^-$ in DMSO-d6/H_2O (200:1)

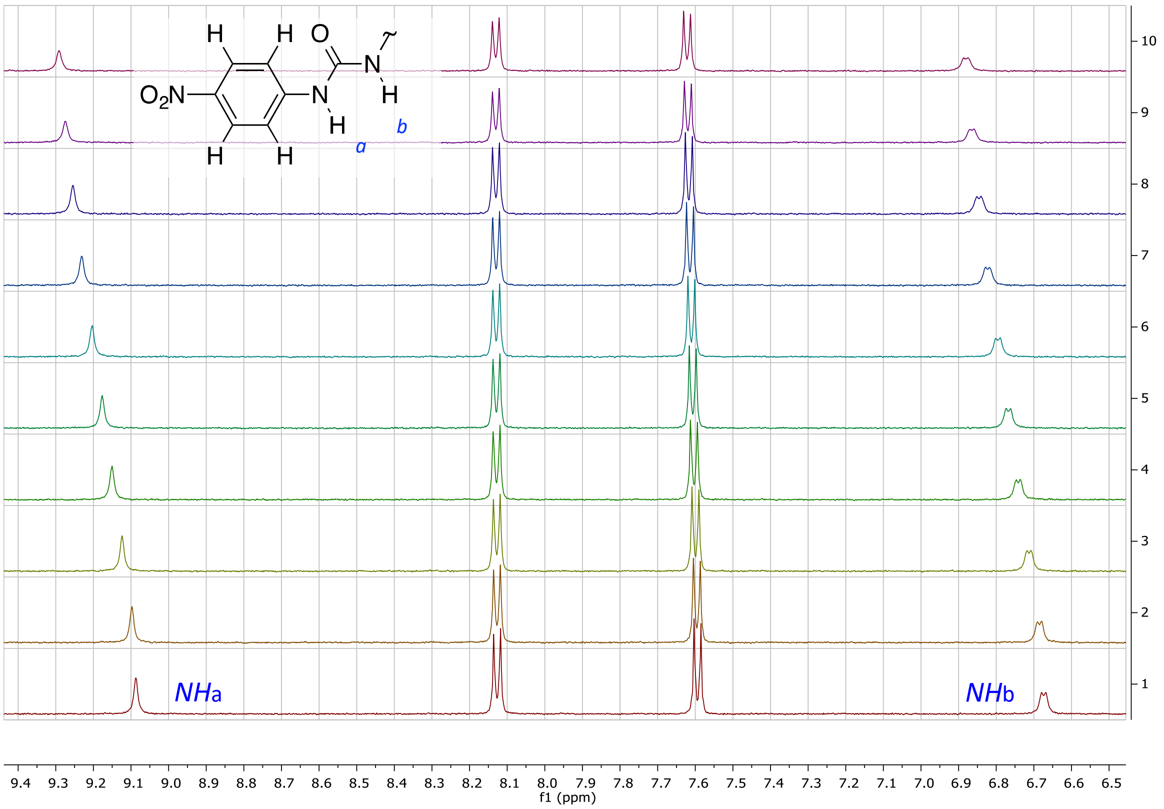

Figure S19. Downfield region of the ^1H NMR spectra from the titration of $Bu_4N^+NO_3^-$ into 1 mM **1** in DMSO with 0.5% H_2O.

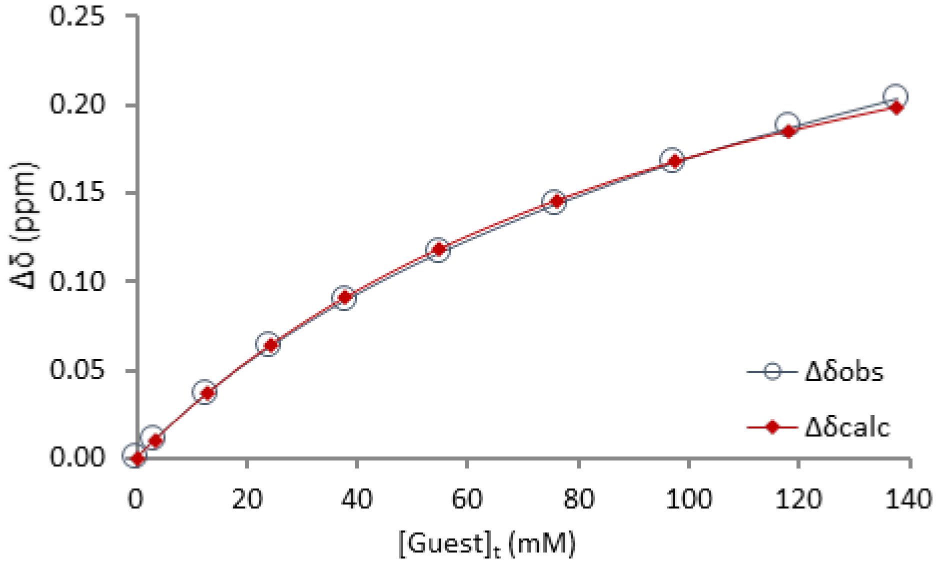

Figure S20. Graph showing the observed binding curve (for NH_a) and calculated fitting for **1** when titrated against $Bu_4N^+NO_3^-$ at 298 K. The movements of both NH signals were used to find $K_a = 9 \pm 0.2\ M^{-1}$.

^1H NMR titration of 1 with $Et_4N^+HCO_3^-$ in DMSO-d6/H_2O (200:1)

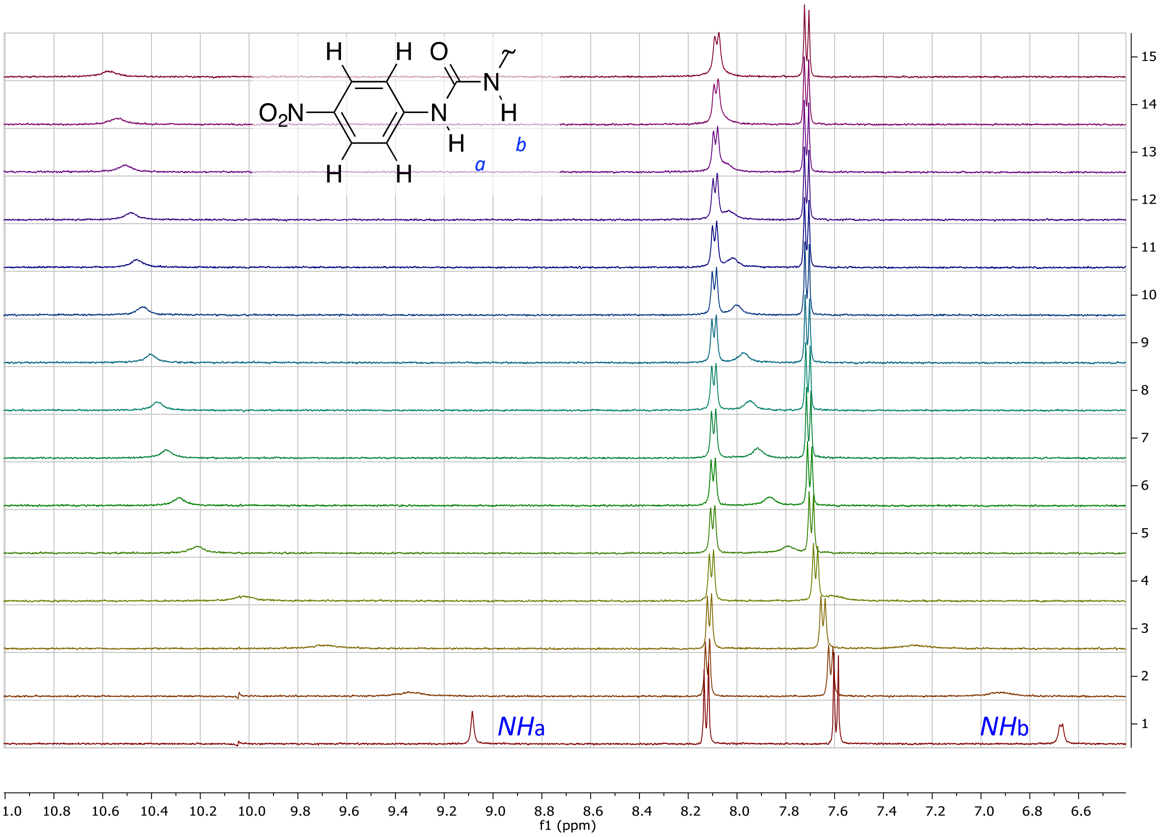

Figure S23. Downfield region of the ^1H NMR spectra from the titration of $Et_4N^+HCO_3^-$ into 1 mM **1** in DMSO with 0.5% H_2O.

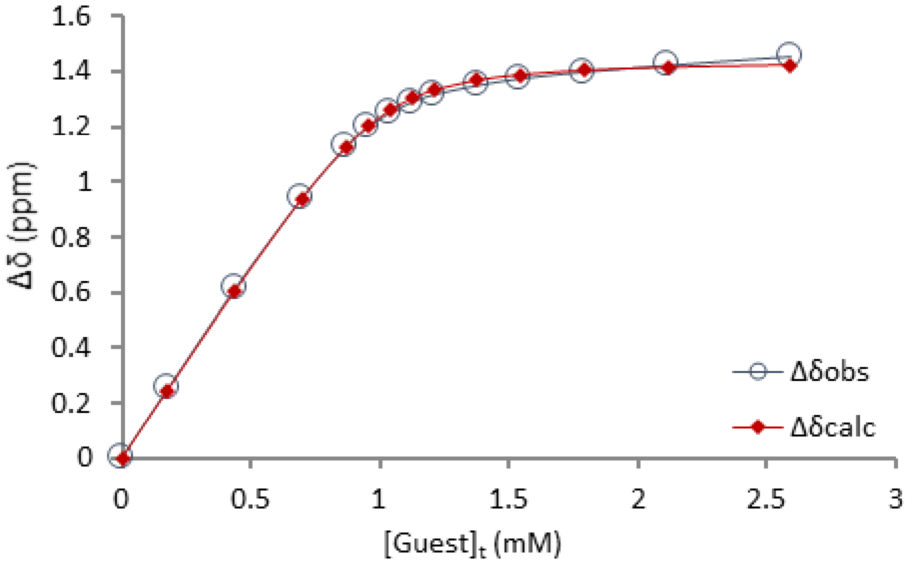

Figure S24. Graph showing the observed binding curve (for NH_a) and calculated fitting for **1** when titrated against $Et_4N^+HCO_3^-$ at 298 K. The movements of both NH signals were used to find $K_a = 41500 \pm 400\ M^{-1}$.

^{1}H NMR titration of 1 with $Bu_4N^+H_2PO_4^-$ in DMSO-d6/H_2O (200:1)

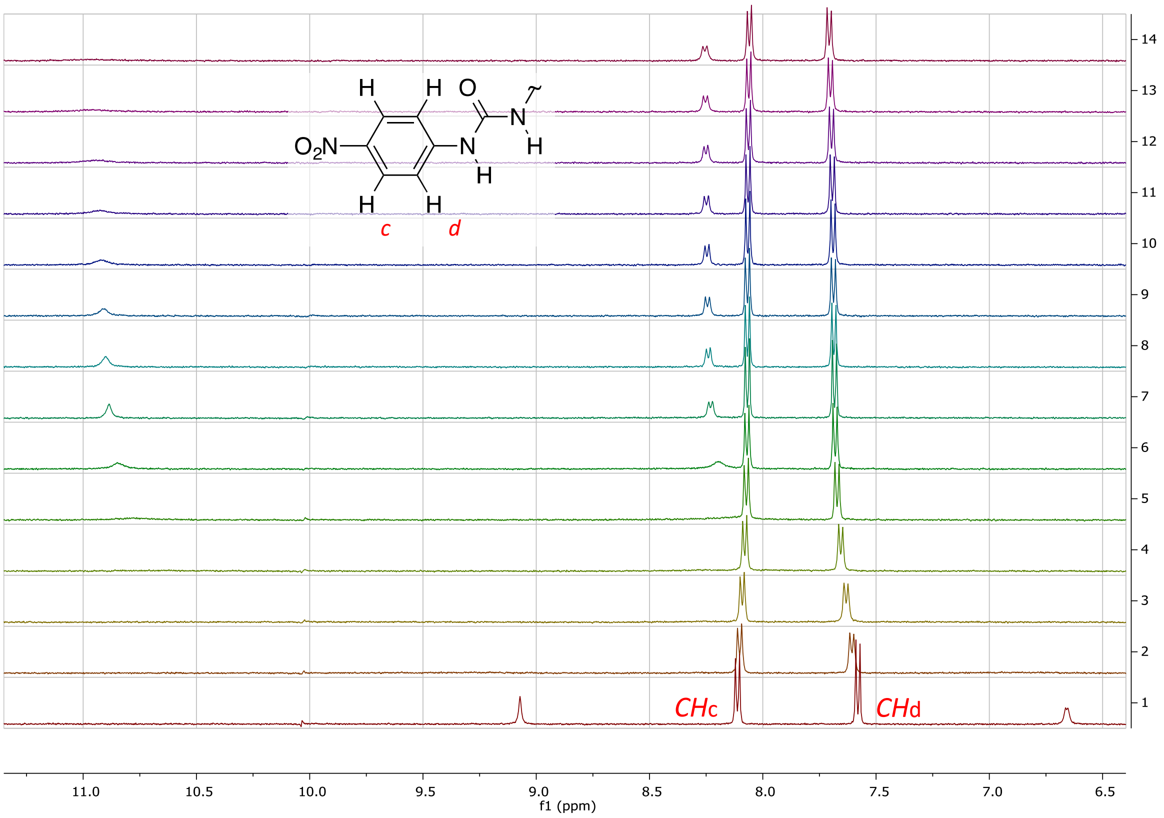

Figure S25. Downfield region of the ^{1}H NMR spectra from the titration of $Bu_4N^+H_2PO_4^-$ into 1 mM **1** in DMSO with 0.5% H_2O.

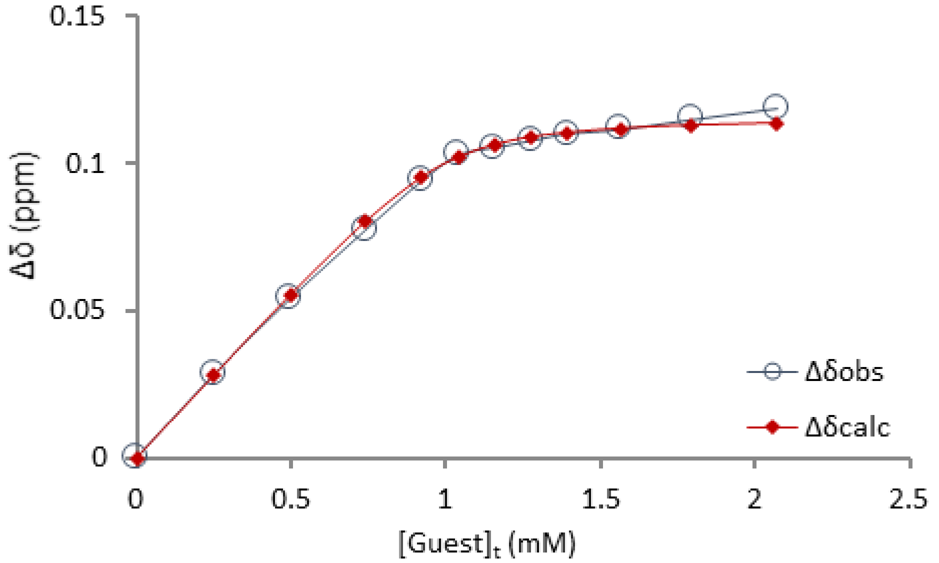

Figure S26. Graph showing the observed binding curve (for CH_d) and calculated fitting for **1** when titrated against $Bu_4N^+H_2PO_4^-$ at 298 K. The movements of both aromatic signals and the 2,7-CH signal were used to find K_a = 45500 ± 1500 M^{-1}.

^{1}H NMR titration of 1 with $Bu_4N^+HSO_4^-$ in DMSO-d6/H_2O (200:1)

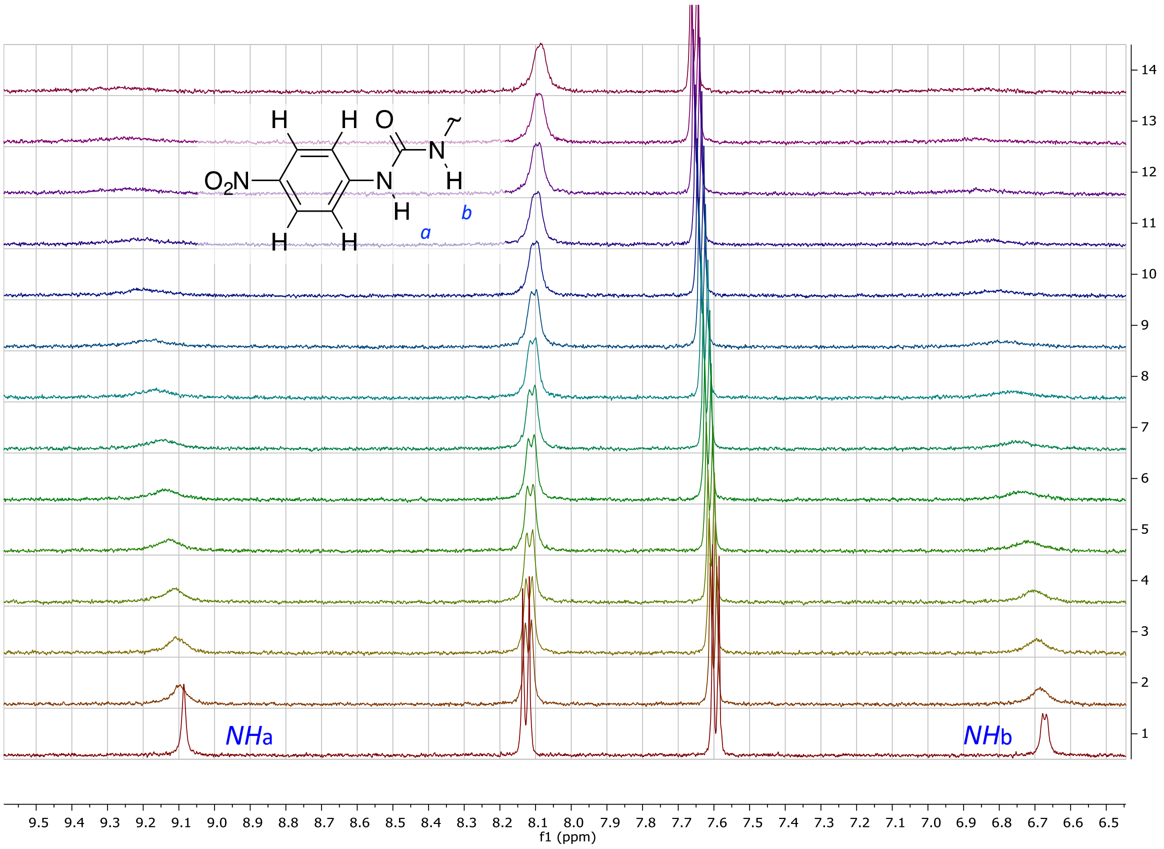

Figure S27. Downfield region of the ^{1}H NMR spectra from the titration of $Bu_4N^+HSO_4^-$ into 1 mM **1** in DMSO with 0.5% H_2O.

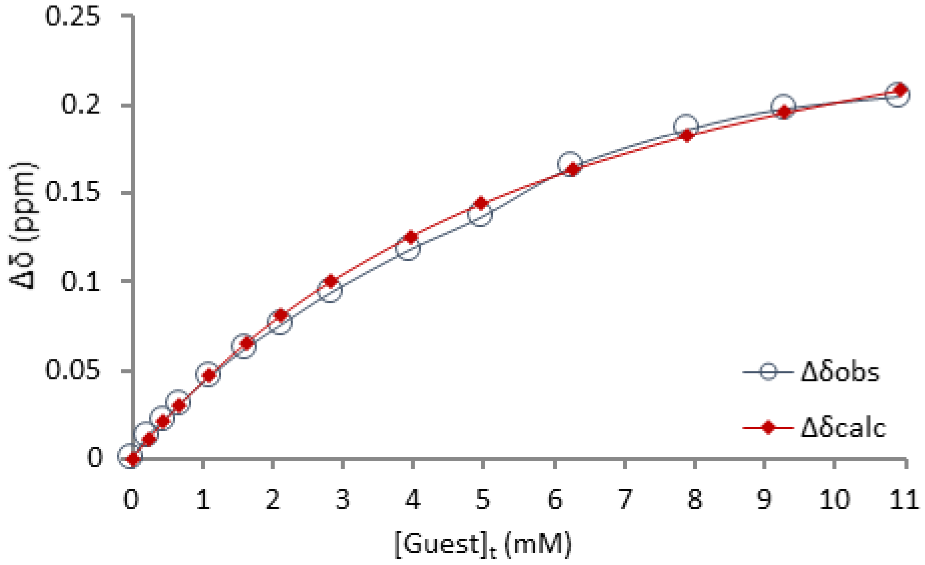

Figure S28. Graph showing the observed binding curve (for NH_b) and calculated fitting for **1** when titrated against $Bu_4N^+HSO_4^-$ at 298 K. The movements of both NH signals were used to find K_a = 179 ± 18 M^{-1}.

^1H NMR titration of 1 with $(Bu_4N^+)_2SO_4^{2-}$ in DMSO-d6/H_2O (200:1)

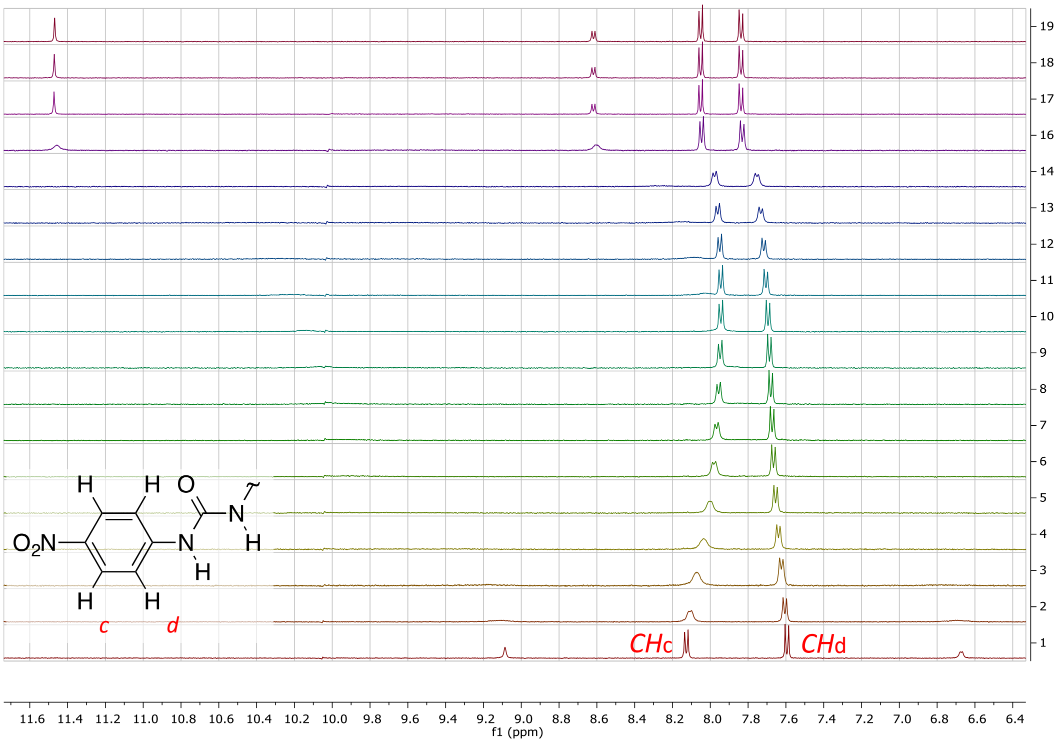

Figure S29. Downfield region of the ^1H NMR spectra from the titration of $(Bu_4N^+)_2SO_4^{2-}$ into 1 mM **1** in DMSO with 0.5% H_2O.

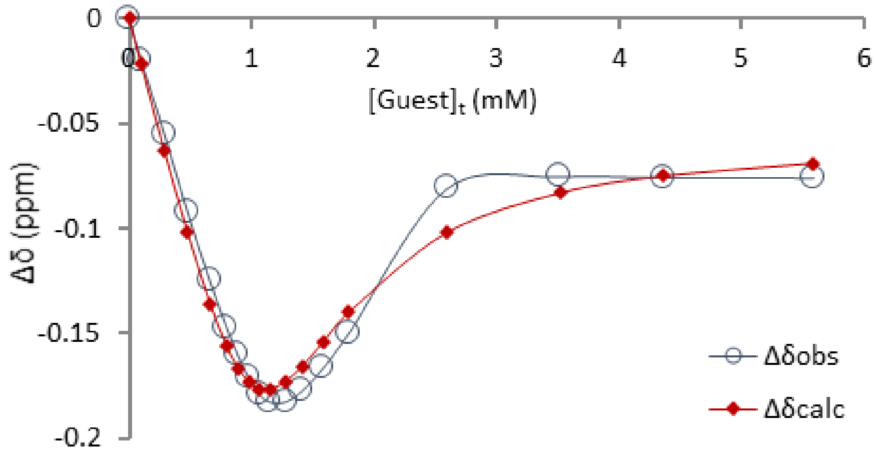

Figure S30. Graph showing the observed binding curve (for $ArCH_c$) and calculated fitting for **1** when titrated against $(Bu_4N^+)_2SO_4^{2-}$ at 298 K. The movements of both aromatic signals and the 2,7-CH signal were fitted to a 1:2 model to find a first $K_a = 1 \times 10^5\ M^{-1}$ and a second $K_a = 3 \times 10^3\ M^{-1}$.

4 References

1. Bruker, *SAINT+ Integration Engine, Data Reduction Software, Bruker Analytical X-ray Instruments Inc., Madison, WI, USA*, 2007.
2. Bruker, *SADABS, Bruker AXS area detector scaling and absorption correction, Bruker Analytical X-ray Instruments Inc., Madison, Wisconsin, USA*, 2001.
3. O. V. Dolomanov, L. J. Bourhis, R. J. Gildea, J. A. K. Howard and H. Puschmann, *J. Appl. Crystallogr.*, **2009**, *42*, 339-341.
4. L. Palatinus and G. Chapuis, *J. Appl. Crystallogr.*, **2007**, *40*, 786-790.
5. L. Palatinus, S. J. Prathapa and S. van Smaalen, *J. Appl. Crystallogr.*, **2012**, *45*, 575-580.
6. G. M. Sheldrick, *Acta Crystallogr., Sect. A: Found. Crystallogr.*, **2008**, *64*, 112-122.
7. G. M. Sheldrick, *Acta Crystallogr. C*, **2015**, *71*, 3-8.
8. Bruker AXS BV, Madison, WI, USA; 1997–2004.
9. Z. Otwinowski, W. Minor, *Methods Enzymol.* **1997**, *276*, 307–326.
10. R. H. Blessing, *J. Appl. Cryst.* **1997**, *30*, 421–426.
11. H. Li, H. Valkenier, L. W. Judd, P. R. Brotherhood, S. Hussain, J. A. Cooper, O. Jurček, H. A. Sparkes, D. N. Sheppard, A. P. Davis, *Nat. Chem.* **2015**, *8*, 24-32.
